# Engineering a live-attenuated porcine reproductive and respiratory syndrome virus vaccine to prevent RNA recombination by rewiring transcriptional regulatory sequences

**DOI:** 10.1128/mbio.02350-24

**Published:** 2024-12-23

**Authors:** Liwei Li, Jinxia Chen, Zhengda Cao, Ziqiang Guo, Jiachen Liu, Yanjun Zhou, Guangzhi Tong, Fei Gao

**Affiliations:** 1 Shanghai Veterinary Research Institute, Chinese Academy of Agricultural Sciences, Shanghai, China; 2 College of Veterinary Medicine, Nanjing Agricultural University, Nanjing, China; 3 Jiangsu Co-Innovation Center for the Prevention and Control of Important Animal Infectious Disease and Zoonose, Yangzhou University, Yangzhou, China; Huazhong Agricultural University, Wuhan, Hubei, China; Lanzhou Veterinary Research Institute, Lanzhou, Gansu, China

**Keywords:** RNA recombination, porcine reproductive and respiratory syndrome virus (PRRSV), transcriptional regulatory sequence (TRS), recombination-resistant, vaccine redesign

## Abstract

Recombination is a significant factor driving the evolution of RNA viruses. The prevalence and variation of porcine reproductive and respiratory syndrome virus (PRRSV) in China have been increasing in complexity due to extensive interlineage recombination. When this recombination phenomenon occurs in live vaccine strains, it becomes increasingly difficult to prevent and control PRRSV. Reverse genetic manipulation to engineer a different transcriptional regulatory sequence (TRS) circuit introduces genetic traps into the viral genome that are lethal to recombinant RNA progeny viruses. In this study, major interlineage recombination patterns were identified between lineage 1 (L1) PRRSVs and lineage 8 (L8) PRRSVs in China, from 2019 to 2023. The recombinant mutant virus, vA-TRSall, was constructed and successfully rescued by rewiring the entire TRS circuit without changing the amino acid-coding sequence in the genome of the PRRSV live vaccine strain vHuN4-F112. The vA-TRSall, with a brand new TRS circuit, provided effective immune protection against the highly pathogenic L8 PRRSV (vHuN4) and epidemic NADC30-like L1 PRRSV (vZJqz21). Recombination analysis *in vitro* and *in vivo* showed that, compared with the vHuN4-F112 and vZJqz21 co-infection groups, the incidence rates of mutation breakpoints and template-switching recombination in the vA-TRSall and vZJqz21 co-infected groups were effectively reduced. The results have enriched our understanding of the critical role of TRS circuits in PRRSV recombination mechanisms and indicate a successful redesign that can endow PRRSV live vaccines with recombination-resistant capabilities.

**IMPORTANCE:**

Porcine reproductive and respiratory syndrome viruses (PRRSVs) are genetically diverse, and this is due in part to their extensive recombination. Live vaccines are widely used to prevent and control PRRS in China. However, owing to the wide variety of live vaccines, non-standard use, and the wild viruses prevalent on pig farms, new strains, generated via RNA recombination, are continuously emerging. Vaccine strains are also involved in PRRSV recombination, which leads to the emergence of new variants and alterations in virulence and pathogenesis. A recombination-resistant genome was engineered by rewiring the entire transcriptional regulatory sequence (TRS) circuit of the live PRRSV vaccine strain. Theoretically, after clinical application, once the virus recombines with the genome of the epidemic strain, the base pairing between the two sets of TRS circuits should be disrupted, resulting in a fatal genetic trap for the generation of an RNA recombinant progeny virus. Therefore, the remodeled PRRSV TRS mutant generated in this study can serve as a recombination-resistant platform for the rational design of safe PRRS vaccines in the future.

## INTRODUCTION

RNA recombination is ubiquitous in RNA viruses and important for viral evolution and immune escape (1). Poliovirus type 1 was the first virus found to undergo RNA recombination, and most RNA recombination phenomena, including that of some picornaviruses, have been found to occur during positive-strand virus replication (2). Template switching is a common recombination mechanism that requires both an RNA-dependent RNA polymerase (RdRp) and RNA template. Three types of RNA molecules are involved: donors, receptors, and the resulting recombinant molecules. The donor RNA is first used as a template to synthesize a new strand. The RdRp detaches from the template, and the newly synthesized strand is then transferred to the receptor template along with the RdRp to continue strand synthesis. During this process, RdRp must be proximal to the template, and there is a special secondary structure on the template that allows RdRp to switch between templates. The RdRp of the brome mosaic virus stops at a position rich in the AU sequence on the template and jumps to another template during replication (3). During this recombination process, the newly synthesized strand acts as a primer. The main factors affecting this recombination mode are the structure of the recombinant molecules and the conditions and ability of RdRp to jump to another template (4).

Porcine reproductive and respiratory syndrome (PRRS) continues to pose a serious threat, as it can have an economically significant impact on the global swine industry. Clinically, a modified live vaccine (MLV) is widely used to prevent and control PRRS in China (5). Two species of porcine reproductive and respiratory syndrome virus (PRRSV), Betaarterivirus suid 1 (PRRSV-1) and Betaarterivirus suid 2 (PRRSV-2), exist in China. Since PRRSV was first discovered in China in 1996, PRRSV-2 has been the main circulating strain (6). Owing to the significant genetic variations in the intragenotypes of the PRRSVs, PRRSV-2 is classified into nine lineages, lineages 1–9 (L1–L9), based on their ORF5 genes (7). In recent years, recombination between wild-type PRRSVs has occurred frequently, especially between L1 and L8 or L3 and L8 PRRSVs (8, 9). Recombination between the wild-type PRRSV and MLV strains has also been reported (10, 11). The recombination of PRRSVs alters viral pathogenicity and transmissibility in pigs, increases difficulties in clinical virus detection, enhances the risk of live vaccines, and makes the PRRS epidemic more complex (12, 13). There is currently an urgent need to redesign a safe and effective novel vaccine for PRRS with recombination-resistant capabilities.

The PRRSV genome is approximately 15 kb in length and includes a 5′ untranslated region (UTR), at least 11 open-reading frames (ORF1a, ORF1b, ORF2a, ORF2b, ORF2TF, ORF3, ORF4, ORF5, ORF5a, ORF6, and ORF7), and a 3′ UTR. ORF1a and ORF1b encode pp1a and pp1ab, respectively, which process at least 16 nonstructural proteins (nsps) (14). Among these, nsp9 possesses RdRp activity and has a crucial role in viral genomic replication and transcription complex formation (15), while also contributing to virulence and quasispecies diversity (16, 17). The other ORFs encode eight structural proteins (18). Similarly to most nidoviruses, a series of nested subgenomic (sg) mRNAs are synthesized by discontinuous transcription processes. These sg mRNAs have common 5′ and 3′ ends, which are polycistronic in structure but monocistronic in function (19). This discontinuous PRRSV transcription model is similar to the jumping RNA recombination mechanism. Transcriptional regulatory sequences (TRSs) in the leader (leader-TRS and TRS-L) and downstream coding regions (body-TRS and TRS-B) are important *cis-*acting elements that have key roles in genomic RNA synthesis. The TRSs in the different positions of the PRRSV genome are rich in “AU” bases and relatively conservative. There are corresponding TRS-Bs in front of the different ORFs of the viral structural genes that regulate the transcription of sg mRNAs. Coronavirus TRSs contain 7–18 nt, whereas arterivirus TRSs contain only 5–8 nt (20). The TRS *cis-*acting element contains not only the core 5–8 nt oligonucleotides but also the flanking sequence and local secondary structure. Together, they form a TRS cassette, just like the components in an “electric circuit,” and are referred to as a TRS circuit (14). The negative-strand extension model is generally accepted for the unique discontinuous sg mRNA transcription of PRRSV. Discontinuous jumps occur in negative-strand synthesis, and the base pairing of TRS-L and TRS-B plays an undoubtedly important role (21). If this base pairing is destroyed, the discontinuous transcription process will be seriously affected, and the proliferation of PRRSV will be reduced or even blocked.

Based on the above mechanism, the core oligonucleotides and the positions and functions of a series of TRSs in the genome of the PRRSV live-attenuated vaccine strain HuN4-F112 were systematically analyzed in this study. The whole TRS circuit of the PRRSV was then rewired using reverse genetic manipulation to engineer a recombination-resistant genome. The results will help to enhance the safety and stability of future live PRRSV vaccine redesigns.

## RESULTS

### Recombination between L1 and L8 PRRSVs is the main pattern in China

**Fig 1:**
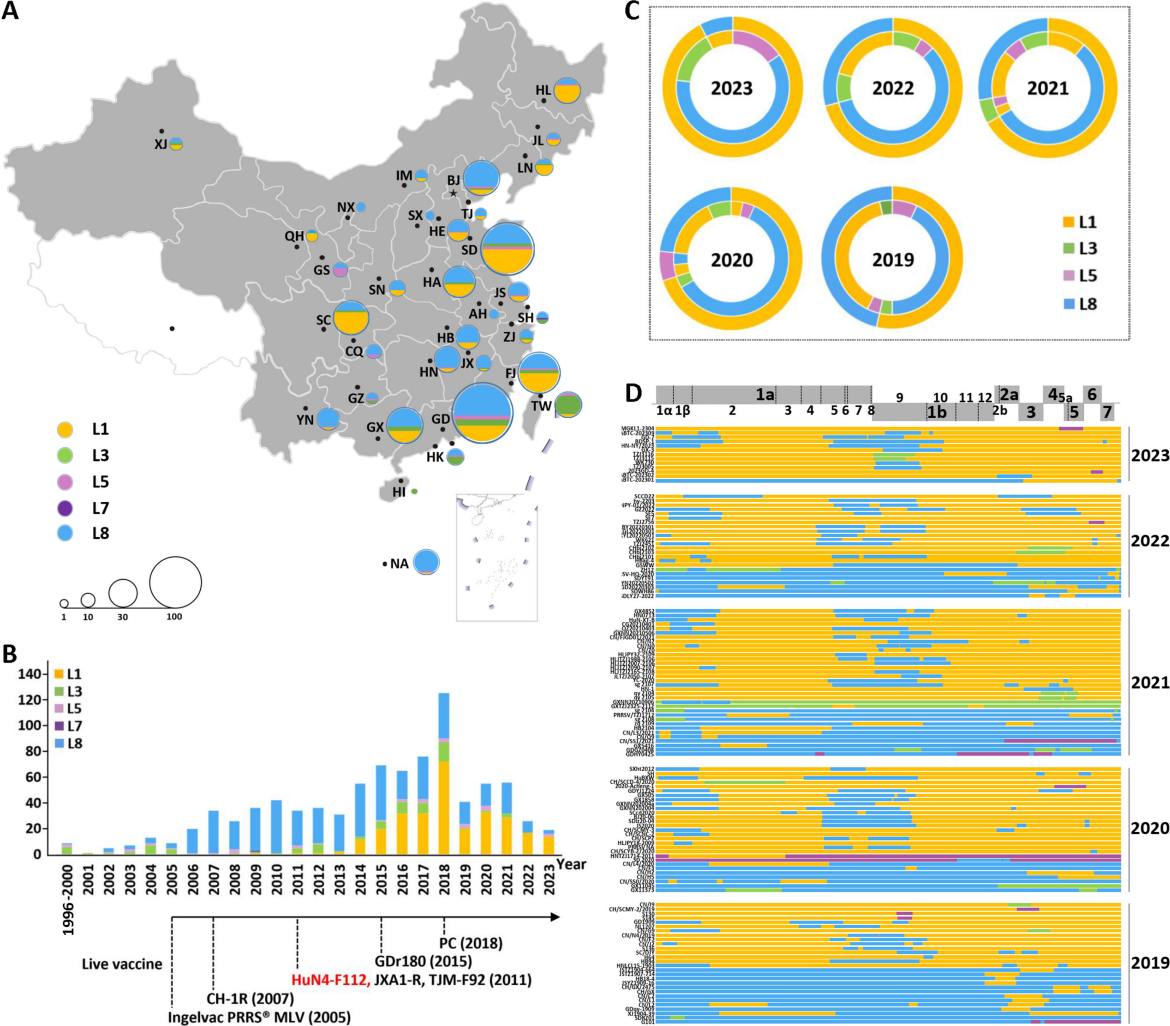
Lineage distribution and recombination patterns of PRRSV-2 in China. (**A**) Geographical distribution of the 893 PRRSV-2 genomes in China. Different lineages are identified by different colors, and each lineage was counted and illustrated using pie charts for each province. The pie sizes represent the numbers of PRRSVs in the 32 provinces of China. (**B**) Temporal distribution of the 893 PRRSV-2 genomes and commercial live vaccines in China. The annual number of each lineage from 1996 to 2023 and the year of live vaccine approval in China are shown. (**C**) Major and minor parental strain contributors in recombinants observed in China from 2019 to 2023. The major parent is shown on the outer ring, and the corresponding minor parent is shown on the inner ring. (**D**) A map of parental lineages of genomes and interlineage recombination patterns in China from 2019 to 2023. A schematic diagram of the full-length genome is displayed, in which the positions and boundaries of the major ORFs and nsps within ORF1a and ORF1b are shown. Each strain name is displayed on the left, and the corresponding major parent is identified in the different lineage colors on the right. A change in color indicates that the region had been replaced by a minor parent, indicating the presence of a recombinant.

To provide insights into the epidemic PRRSVs in China, 893 complete sequences of PRRSV-2 strains, collected from 32 Chinese provinces from 1996 to 2023, were downloaded from the GenBank database (Table S1). The geographical distributions for the different lineages in each province were counted and illustrated by different colors (Fig. 1A). Strains from Guangdong, Shandong, and Fujian accounted for 13%, 11%, and 9% of the Chinese PRRSVs, respectively. The year-to-year distributions for the different lineages and the year of approval for the live PRRS vaccines in China were also investigated (Fig. 1B). The results showed that the dominant lineage between 1991 and 2005 was L3 PRRSV, but after the 2006 breakout of highly pathogenic PRRSV (HP-PRRSVs), L8 PRRSV became the dominant lineage. With the emergence of NADC30- and NADC34-like PRRSVs in 2013, however, the proportion of L1 PRRSV gradually increased and became the dominant lineage, although L5 and L8 PRRSVs were also abundant and widely distributed in numerous countries. According to the year of live vaccine approval in China, there has not been a vaccine for the current dominant lineage, and the main vaccines are still derived from L8 PRRSVs, such as HuN4-F112, the vaccine strain used in this study (Fig. 1B). To explore the current recombination characteristics of PRRSVs in China, the major and minor parental strain contributors to recombinants were determined from 2019 to 2023. The major recombination patterns for this period were L1 PRRSV (major parent) and L8 PRRSV (minor parent), and L8 PRRSV (major parent) and L1 PRRSV (minor parent; Fig. 1C). The locations of the recombination breakpoints were analyzed according to the position of VR-2332 in the genome and ranged from relative nucleotide positions of approximately 6,500–8,600 (in the nsp5 to nsp9 region) and 11,900–14,200 (in the GP2 to GP5 region; Fig. 1D; Table S2). The analysis indicates that L1 PRRSV has the highest recombination proportion among all individual lineages, and the proportion of recombination based on the L1 backbone increased annually, especially for L8 PRRSV, which is currently used in live vaccines against PRRSV.

### Only whole TRS circuit mutation produces infectious progeny virus with parental virus characteristics

**Fig 2:**
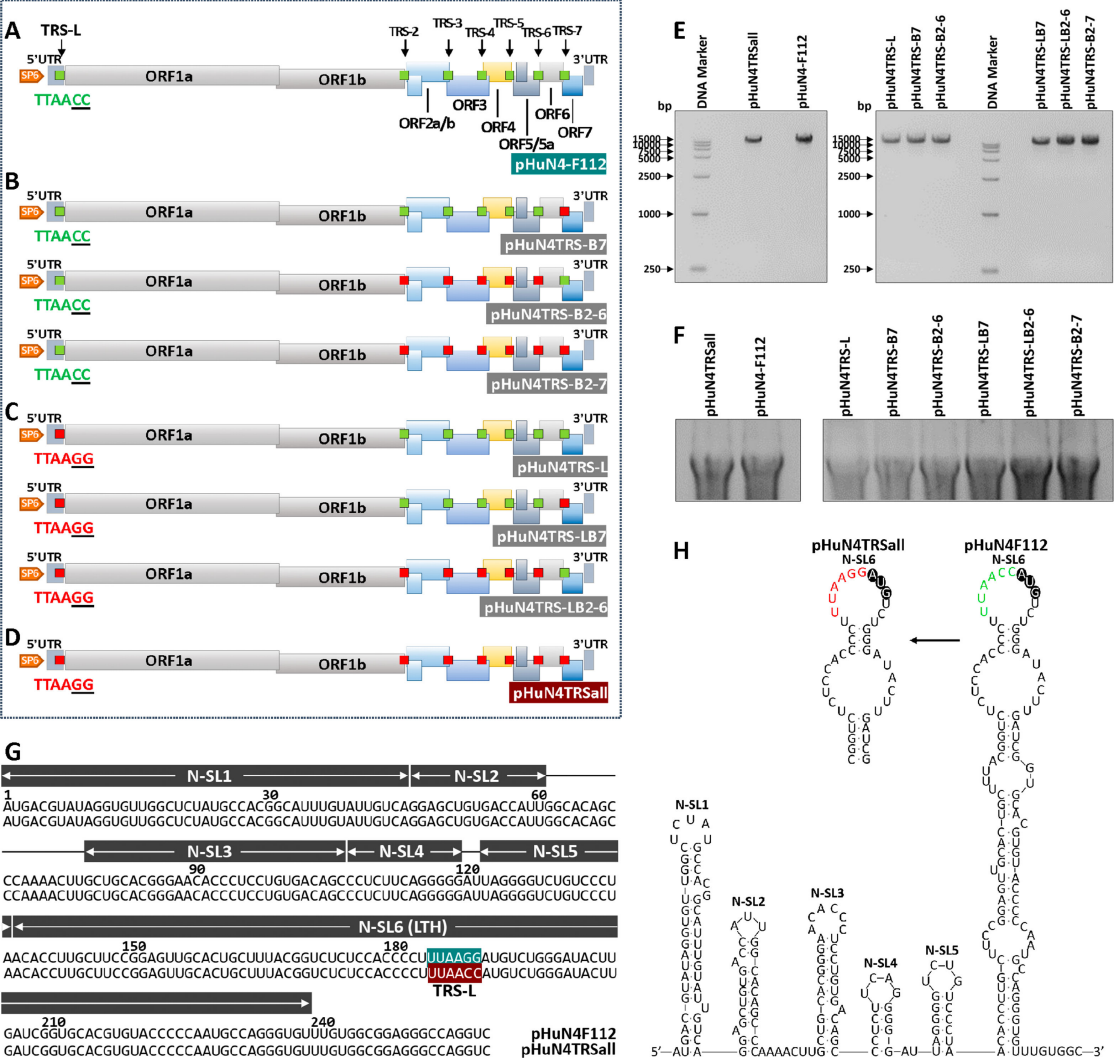
Construction strategy and secondary structure predictions of mutant viruses containing redesigned TRS circuits. (**A–D**) Schematic diagram of the TRS cassette series chimeric or whole mutant full-length PRRSV infectious clone construction. The squares represent the leader-TRS (TRS-L), TRS-2, TRS-3, TRS-4, TRS-5, TRS-6, and TRS-7 according to the position in the genome. Red indicates “C_5_C_6_” mutated to “G_5_G_6_” in the core hexabases, and green indicates not mutated. (**E**) Identification of the corresponding linearized templates for *in vitro* transcription by nucleic acid electrophoresis. The length of the linearized template of the full-length PRRSV infectious clone and pBluescript SK II (+) vector is 18,304 bp. (**F**) Identification for the corresponding *in vitro* transcription RNAs by nucleic acid electrophoresis. (**G**) The nucleotide sequence alignment of the 5′-proximal 260 bp region between pHuN4-F112 and pHuN4TRSall by Clustal X. Nucleotides of the conserved domains (N-SL1, N-SL2, N-SL3, N-SL4, N-SL5, and N-SL6) are shaded gray. The TRS-L and body-TRS are shaded in green and red, respectively. (**H**) RNA secondary structures of the 5′-proximal 260 bp region of PRRSV genomes, as predicted by Mfold. N-SL1-5 denotes the stem–loop structures that exist in the 5′ UTR. The LTH was contained in N-SL6. The TRS-L is shaded green and red, respectively, and the ORF1 translational initiation codon is shown in black. The “C_5_C_6_” to “G_5_G_6_” mutation does not affect the LTH structure.

To enhance the recombination resistance capability of the current commercial vaccine strain HuN4-F112, a series of chimeric or whole TRS circuit mutations based on the full-length PRRSV infectious clone were constructed according to a schematic diagram (Fig. 2A through D). Green squares in Fig. 2A represent the natural TRS-L and TRS-B (TRS-2, TRS-3, TRS-4, TRS-5, TRS-6, and TRS-7) according to their position in the genome of pHuN4-F112. Red squares in Fig. 2B represent the chimeric mutations in TRS-B that generate pHuN4TRS-B7, pHuN4TRS-B2-6, and pHuN4TRS-B2-7. Red squares in Fig. 2C represent the chimeric mutations in TRS-L and TRS-B used to generate pHuN4TRS-L, pHuN4TRS-LB2-6, and pHuN4TRS-LB7. The entire TRS cassette mutation shown in Fig. 2D causes TRS circuit rewiring to conduct pHuN4TRSall. The results of nucleic acid electrophoresis showed that the corresponding linearized templates and *in vitro* transcription RNAs were consistent with those of the parental pHuN4-F112 (Fig. 2E and F). As shown in Fig. 2G and H, the conserved domains and RNA secondary structures (N-SL1, N-SL2, N-SL3, N-SL4, N-SL5, and N-SL6) were not affected by the TRS circuit rewiring. Furthermore, the “C^_5_^C^_6_^” to “G^_5_^G^_6_^” mutation did not affect the leader TRS hairpin (LTH) structure between pHuN4TRSall and pHuN4-F112.

**Fig 3:**
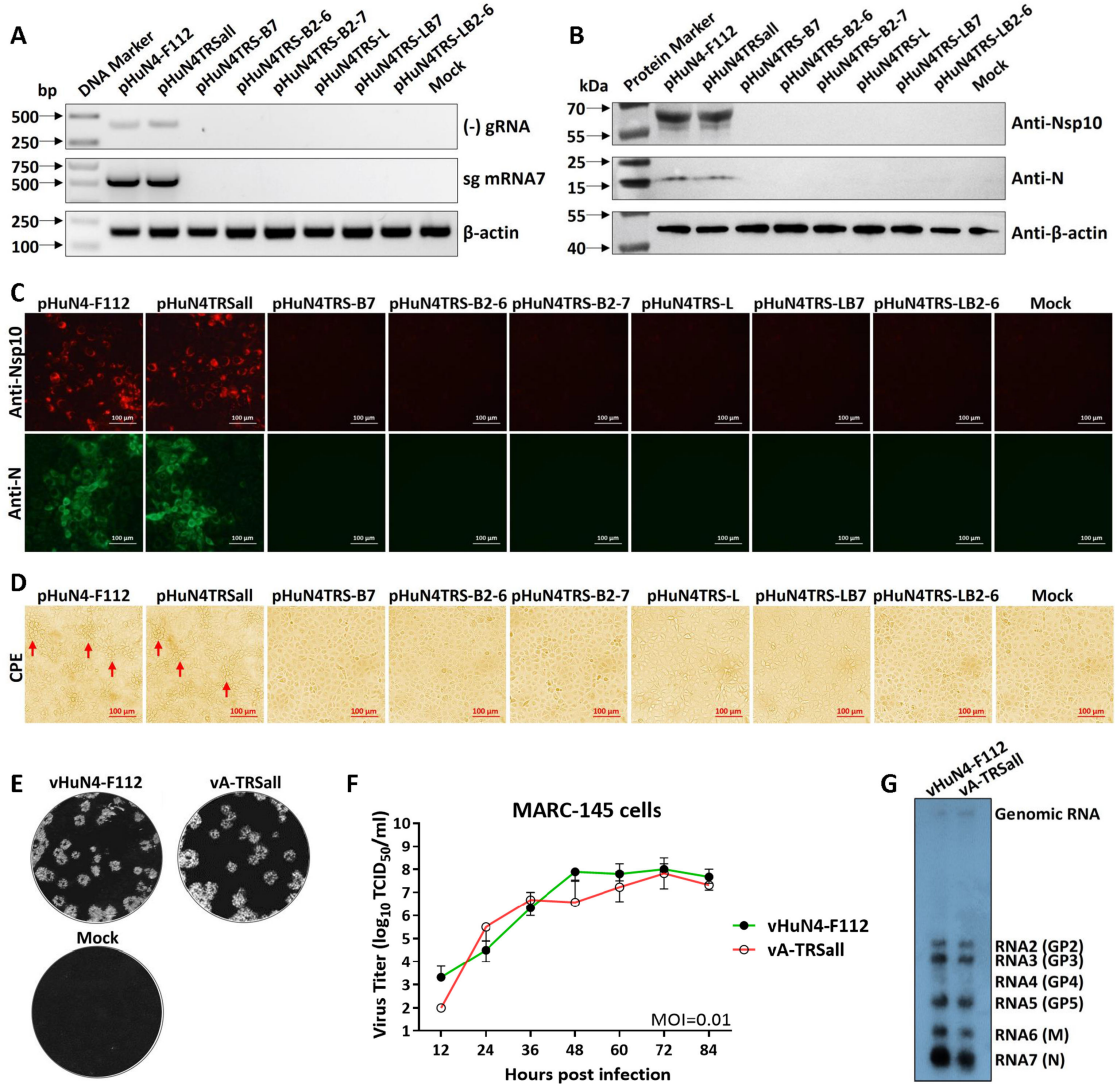
Analysis of RNA transcription, protein translation, and virological characteristics of the mutant viruses containing redesigned TRS circuits. (**A**) The PCR products of (−) gRNA, sg mRNA7, and β-actin were determined by electrophoresis. β-actin was used as an internal control. (**B**) The expression of PRRSV non-structural protein nsp10 and structural protein N was determined by WB, with β-actin used as a loading control. (**C**) The expression of PRRSV nsp10 and N protein was determined by IFA. Cells were stained with anti-mouse Alexa-568-labeled (red) or anti-mouse Alexa-488-labeled (green) secondary antibodies, respectively. The immunofluorescence was visualized using an inverted fluorescence microscope. Scale bar = 100 µm. (**D**) The CPEs were observed in MARC-145 cells inoculated with supernatants collected from TRS cassette mutant-transfected BHK-21 cells. Scale bar = 100 µm. (**E**) Comparison of plaque morphology and number between the vA-TRSall and vHuN4-F112. The MOI of 0.01 were chosen for infecting fresh MARC-145 cells, and the plaques were stained with crystal violet at 4 days post-infection. (**F**) Comparison of viral growth kinetics between vA-TRSall and vHuN4-F112 in MARC-145 cells. Cells were infected at MOI of 0.01, and the supernatants were harvested at indicated time points, followed by viral titration. Data represent the mean ± SD of three independent experiments. Statistical significance was analyzed using *t* tests. (**G**) Comparison of viral RNA transcription levels between vA-TRSall and vHuN4-F112 by northern blotting at an MOI of 1. Intracellular RNAs were extracted at 36 hpi. The RNAs were separated on denaturing 1% agarose gels and blotted onto a nitrocellulose membrane, and sg mRNAs 2–7 were labeled.

Transfection rescue was performed in three parallel experiments on the above mutant clones and parental pHuN4-F112. For the detection of negative-strand gRNA [(−) gRNA] by reverse transcription-PCR (RT-PCR) and sg mRNA7 by leader–body junction PCR, the levels of viral (−) gRNA and sg mRNA7 synthesis of the pHuN4TRSall showed no difference from those of pHuN4-F112, while the viral (−) gRNA and sg mRNA7 were not detected in the other six chimeric mutant clones (Fig. 3A). The expression levels of the non-structural protein nsp10 and structural protein N were assessed using western blotting (WB) and indirect immunofluorescence assay (IFA). The results showed that only pHuN4TRSall and pHuN4-F112 had specific protein expression with similar characteristics, and no bands or fluorescence results were detected for the other six chimeric mutant clones (Fig. 3B and C). Similar results were observed for the cytopathic effect (CPE), as only pHuN4TRSall and pHuN4-F112 were found to have the typical CPE of PRRSV (Fig. 3D). After three additional blind passages, six TRS cassette chimeric mutant clones could not be successfully rescued. The above results suggest that only TRS cassettes whole mutation produced the infectious progeny virus, named vA-TRSall. There was no significant difference in terms of plaque morphology and number for the mutant virus vA-TRSall when compared to the parental virus vHuN4-F112 (Fig. 3E). After inoculation with the mutant or parental viruses at multiplicity of infection (MOI) of 0.01, the virus supernatants of the infected cells were collected at different time points, virus titers were then measured, and a multi-step growth curve was drawn for comparison. The results showed that the titers of mutant virus vA-TRSall were slightly lower 12 h post-infection (hpi) than those of the parental virus vHuN4-F112. Both vA-TRSall and vHuN4-F112 reached peak replication at 72 hpi and then decreased and stabilized (Fig. 3F). To analyze the sg mRNA profiles of MARC-145 cells infected with the mutant viruses, the total intracellular RNA was extracted from the virus-infected cell lysates for northern blotting. The results showed that the sg mRNAs of the parental vHuN4-F112 and mutant vA-TRSall both reacted with the PRRSV-specific probe. Sg mRNAs 2, 3, and 4 have a similar amount (GP2/3/4 form the trimer on the virion), and sg mRNA7 was most abundant during the viral transcription process, as shown in Fig. 3G. The results suggest that growth characteristics of the mutant virus after TRS circuit redistribution were not significantly different from those of the parental virus vHuN4-F112, indicating that the TRS circuit rewiring does not affect the replication and transcription of PRRSV in permissive cell lines.

### Mutant virus with TRS circuit rewiring prevents the recombination with NADC30-like PRRSV *in vitro*

**Fig 4:**
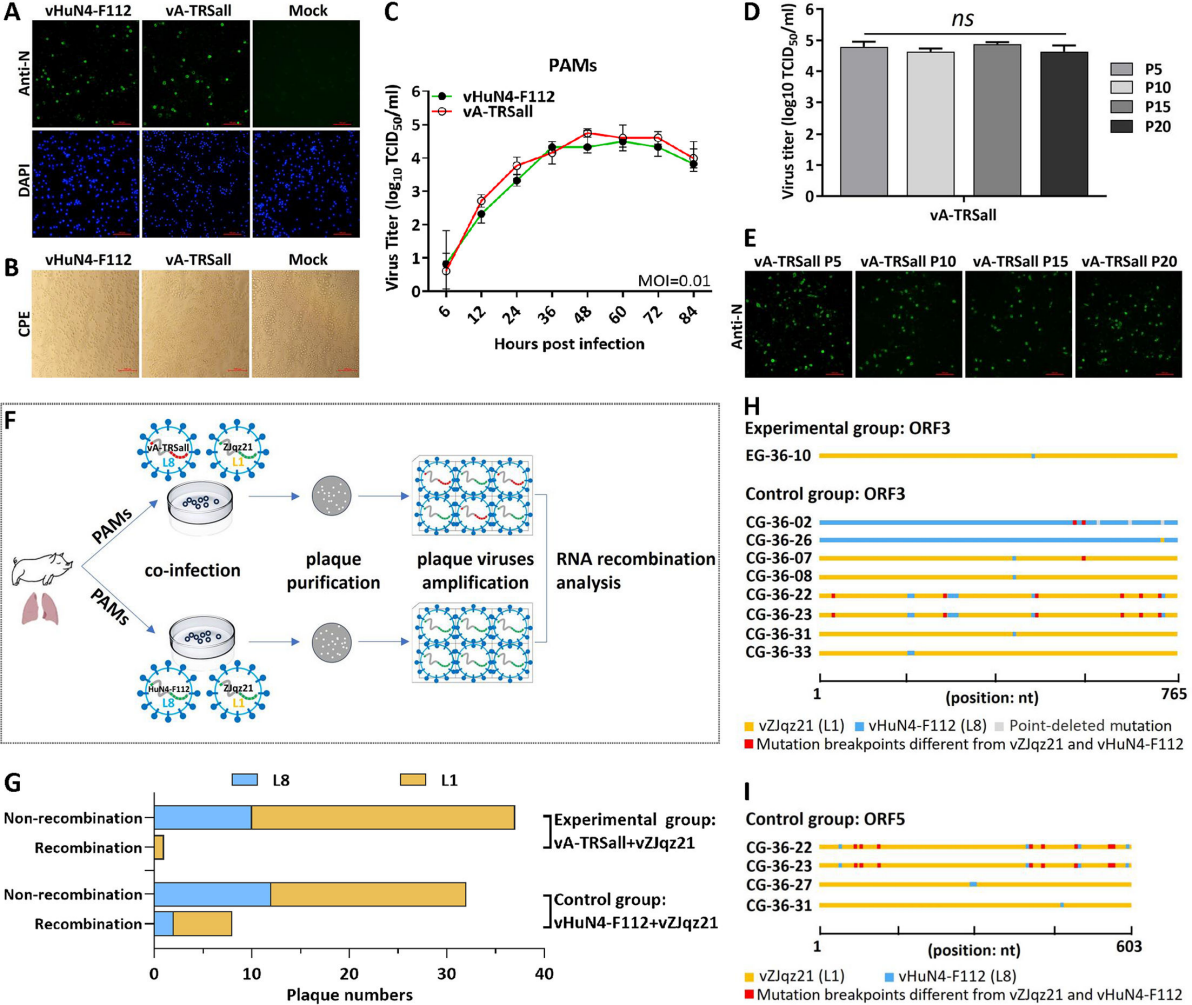
Recombination analysis of *in vitro* co-infection in PAMs. (**A**) The expression of PRRSV N protein was determined in PAMs infected by vA-TRSall or vHuN4-F112 (MOI = 0.01) at 48 hpi. Cells were stained with anti-mouse Alexa-488-labeled (green) secondary antibodies. Cell nuclei were stained with 4',6'-diamidino-2-phenylindole. The immunofluorescence was visualized using an inverted fluorescence microscope. Scale bar = 100 µm. (**B**) The CPEs were observed in PAMs infected by vA-TRSall or vHuN4-F112 (MOI = 0.01) at 48 hpi. (**C**) Comparison of viral growth kinetics between vA-TRSall and vHuN4-F112 in PAMs. Cells were infected at MOI of 0.01, and the supernatants were harvested at indicated time points, followed by TCID_50_ measurement. Data represent the mean ± SD of three independent experiments. Statistical significance was analyzed using *t* tests. (**D and E**) Detection of virus titers and N protein expression in vA-TRSall (P5, P10, P15, and P20 viral stocks) by TCID_50_ measurement or IFA detection using anti-N polyclonal antibody as the primary antibody. Cells were infected at MOI of 0.01 and detected at 48 hpi. Scale bar = 100 µm. (**F**) Schematic of the recombination assay in PAMs. PAMs were harvested from the lungs of 4-week-old PRRSV-negative piglets. The vA-TRSall or vHuN4-F112 was used to co-infect PAMs together with vZJqz21. After co-infection, virus supernatants were collected for plaque purification and sequencing to analyze the chance of recombination. (**G**) The proportion of recombinant PRRSVs in the plaque viruses from the experimental group (vA-TRSall and vZJqz21 co-infection) and control group (vHuN4-F112 and vZJqz21 co-infection). (**H and I**) A map of recombination patterns in ORF3 (**H**) or ORF5 (**I**) of the recombinant plaque viruses. Each plaque virus is displayed on the left, and the corresponding major parent is displayed in different lineage colors on the right. The change in color indicates that the region is identified as a recombinant.

The replication of vA-TRSall (mutant) and vHuN4-F112 (parental) in target cells was investigated using *in vitro* analysis. The N protein expression, CPE, and viral growth kinetics of vA-TRSall were identical to those of vHuN4-F112 in porcine alveolar macrophages (PAMs) during the P5 infection stage (Fig. 4A through C). Serial cell passaging of vA-TRSall was performed to detect the genetic stability of the TRS cassette mutation sites using RT-PCR and nucleotide sequencing. Sequence alignment data showed that the TRS cassette mutation sites of vA-TRSall were stable for at least 20 passages in PAMs. The P5, P10, P15, and P20 viral stocks were then used to determine viral titers in the PAMs. The results showed that the peak titers of different viral stocks remained stable during the passages and were not significantly different (Fig. 4D). Furthermore, IFA using an FITC-conjugated polyclonal antibody against the N protein among different viral stocks was consistent with the viral titer data (Fig. 4E). These results indicate that the mutant virus with TRS circuit rewiring is genetically stable and has no difference in its replication characteristics in target cells when compared with the parental vaccine strain.

Subsequently, co-infection experiments were conducted in target cells to evaluate the recombination resistance capability of vA-TRSall, as shown in the schematic diagram (Fig. 4F). vA-TRSall or vHuN4-F112 was used to co-infect PAMs with the NADC30-like strain vZJqz21 (L1), as reported previously (22). Viral supernatants were collected at 36 hpi for plaque purification and PCR analysis. Sequencing analysis indicated that the amount of recombinant PRRSVs in the plaque viruses from the vA-TRSall group (experimental group) was less than that in the vHuN4-F112 group (control group, CG; Fig. 4G; Table 1). In most recombinants in the vHuN4-F112 group, the major parental strain was vZJqz21, indicating that L1 PRRSVs provide a backbone for most recombination events. The positions of the recombinant breakpoints were distributed throughout the gene region of ORF3 and ORF5 (Fig. 4H). Mutation breakpoints different from those of the vZJqz21 and vHuN4-F112 sequences were produced at several sites in the gene region of ORF3 and ORF5. In the experimental group co-infected with vA-TRSall and vZJqz21, no mutation breakpoint was detected in the gene region of ORF5 (Fig. 4I). Because of the different adaptabilities to PAM, this experiment preliminarily proved that PRRS vaccine strains redistributed by the TRS circuit will effectively reduce the probability of recombination among strains during *in vitro* co-infections when compared with the parental vaccine strains. To further evaluate the recombination resistance of the mutant virus with TRS circuit rewiring, co-infection experiments were performed in piglets.

**TABLE 1:** Putative recombination breakpoints upon co-infection *in vitro^a^*

Representative recombinant virus	Breakpoint number	Position
Experimental group (vA-TRSall and vZJqz21 co-infection)		
ORF5 gene		
None		
ORF3 gene		
EG2-36-10	1	501
Control group (vHuN4-F112 and vZJqz21 co-infection)		
ORF5 gene		
CG-36-22,23	13	57, 101, 109, 181, 387–390, 401, 492, 493, 500, 501, and 599
CG-36-27	2	299 and 300
CG-36-31	1	472
ORF3 gene		
CG-36-02	5	543, 566, 578, 669, and 701
CG-36-26	1	701
CG-36-07	2	401 and 566
CG-36-08,31	1	401
CG-36-22,23	16	32, 194–196, 286–289, 470, 471, 612, 667, and 700–703
CG-36-33	3	194–196

^*a*^ X-Y-Z (X = EGor CG; representing the experimental or control groups, respectively; Y = hpi; Z = serial number of plaque viruses).

### Mutant virus with TRS circuit rewiring still provides good immune protection against HP-PRRSV and NADC30-like PRRSV challenge in piglets

**Fig 5:**
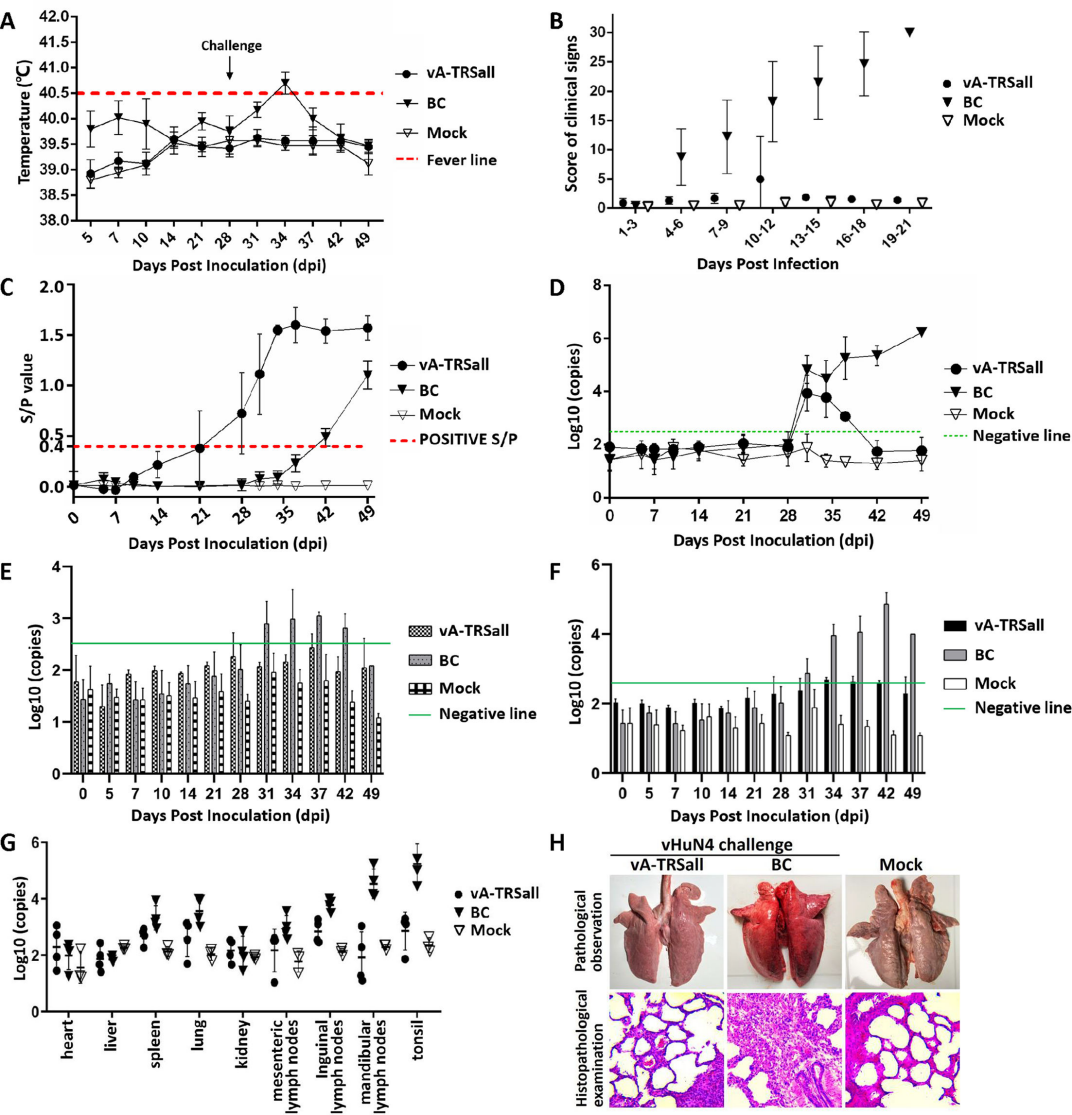
Immune protection of vA-TRSall against HP-PRRSV challenge *in vivo*. (**A**) Temperature monitoring data for the immune challenge (vA-TRSall), BC, and blank control (mock) groups. The red dashed line indicates the fever line. Piglets in the vA-TRSall group and BC group were challenged with vHuN4 (10^4^ TCID_50_) at 28 dpv. (**B**) Clinical symptoms after the immune challenge were monitored and scored in the vA-TRSall, BC, and mock groups. (**C**) The humoral immune response of the PRRSV N protein was detected by enzyme-linked immunosorbent assay (ELISA) (*S*/*P* ratio), and serum samples were collected from the vA-TRSall, BC, and mock groups at specified times. The red dashed line indicates the positive line. (**D**) Detection of viremia changes in the three groups at specified times using RT-qPCR. (**E and F**) Nasal and (**E**) anal (**F**) swab samples from the three groups were collected at specified time points to detect changes in the viral load. (**G**) Determination of the viral load in abrasive fluid samples from different tissues of the three groups at the end of the experiment. (**H**) Histopathology analysis of the lungs from piglets in the three groups.

The mutant virus with TRS circuit rewiring was used in an immune protection test with 6-week-old piglets, and the HP-PRRSV vHuN4 strain challenge was carried out 28 days post-vaccination (dpv), with a total test period of 49 days, as shown in Fig. S1. Temperature and clinical symptoms were monitored. The results showed no significant differences between the immune challenge (vA-TRSall) and mock control (Mock) groups in terms of body temperature changes or clinical sign scores. The blank challenge (BC) group exhibited fever, dyspnea, and a runny nose (Fig. 5A and B). The scores for clinical signs in the BC group from 4 to 21 days post challenge (dpc) were significantly higher than those in the vA-TRSall and mock groups (*P* < 0.001, Fig. 5B). All the collected serum samples were tested for specific antibodies against PRRSV. The results showed that the antibody levels increased to a positive level 21 days after immunization with vA-TRSall. On day 37, after 9 days of the HP-PRRSV vHuN4 challenge, the *S*/*P* value reached its peak and remained stable until 49 dpv (Fig. 5C). Viremia in the vA-TRSall group increased after the vHuN4 challenge and decreased to below the negative line at 14 dpc. There was no upward trend in the later period (Fig. 5D), indicating that the PRRS-attenuated vaccine strain with TRS circuit rewiring could effectively alleviate the typical clinical symptoms and viremia caused by the HP-PRRSV challenge. During the experiment, nasal and anal swab viral loads were also detected, and the results showed that virus shedding on both swabs from the vA-TRSall group was lower when compared with the BC group or the negative line until the end of the experiment (Fig. 5E and F). The numerical values of the viral loads in the tissues showed similar trends to the detection of virus shedding. The viral load in the vA-TRSall group was lower than that in the BC group and was the highest in the mandibular lymph nodes and tonsils (Fig. 5G). The BC group showed typical interstitial pneumonia and collapsed alveoli with the infiltration of numerous inflammatory cells into the alveolar spaces. Neither macroscopic nor histological changes in lung lesions were observed in other two groups (Fig. 5H), indicating that the vA-TRSall vaccination significantly alleviated the clinical signs caused by the homologous L8 PRRSV challenge.

**Fig 6:**
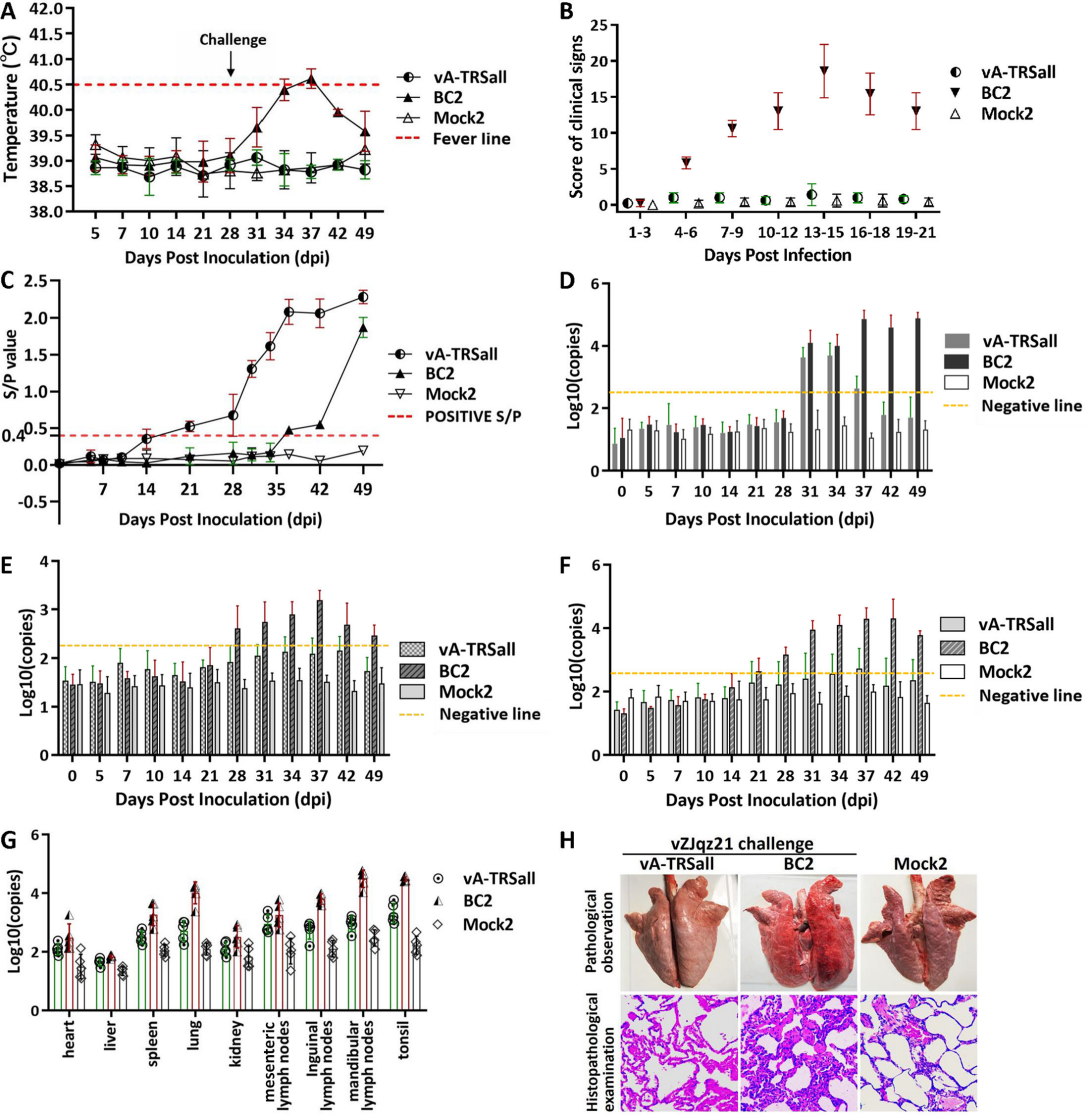
Immune protection of vA-TRSall against NADC30-like PRRSV chanllenge *in vivo*. (**A**) Temperature monitoring data for the immune challenge (vA-TRSall), blank challenge (BC2), and blank control (Mock2) groups. The red dashed line indicates the fever line. Piglets in the vA-TRSall and BC groups were challenged with vZJqz21 (10^5^ TCID_50_) at 28 dpv. (**B**) Clinical symptoms after the immune challenge were monitored and scored for the vA-TRSall, BC2, and Mock2 groups. (**C**) The humoral immune response of the PRRSV N protein was detected by ELISA (*S*/*P* ratio), and serum samples were collected from the vA-TRSall, BC2, and Mock2 groups at specified time points. The red dashed line indicates the positive line. (**D**) Detection of viremia changes in the three groups at the specified time points using RT-qPCR. (**E and F**) Nasal and (**E**) anal (**F**) swab samples from the three groups were collected at specified times to detect changes in the viral load. (**G**) Determination of the viral load in abrasive fluid samples from different tissues of the three groups at the end of the experiment. (**H**) Histopathology analysis from the lungs of piglets in the three groups.

An immune protection test for the mutant virus with a rewired TRS circuit against the NADC30-like PRRSV vZJqz21 strain challenge was performed using methods similar to those used in the above experiments, as shown in Fig. S2. The results showed similar trends to those of the immune protection test against the vHuN4 challenge. The immune challenge (vA-TRSall) and mock control (Mock2) groups had similar characteristics in terms of temperature and clinical scores, whereas the blank challenge (BC2) group had fever and dyspnea from 31 to 34 dpc (Fig. 6A and B). The antibody level increased to a positive level 21 days after immunization with vA-TRSall and gradually increased until 49 dpv (Fig. 6C). Viremia in the vA-TRSall group increased after the vZJqz21 challenge and decreased below the negative line in the later period (Fig. 6D). The level of viral shedding identified in both the nasal and anal swabs of the BC2 group was higher than that in the other two groups (Fig. 6E and F). The viral load was also higher in tissues and organs of the BC2 group (Fig. 6G). The BC2 group displayed typical lung lesions, exfoliated epithelial cells in the bronchioles, and collapsed alveoli with the infiltration of numerous inflammatory cells. Neither macroscopic nor histological changes in lung lesions were observed in other two groups (Fig. 6H). The results indicate that the vA-TRSall vaccination effectively alleviates the clinical signs caused by the heterologous L1 PRRSV challenge, further suggesting that the PRRS-attenuated vaccine strain with TRS circuit rewiring will provide adequate homologous and heterogeneous protection against HP-PRRSV and NADC30-like strains.

### Mutant virus with TRS circuit rewiring reduces the recombination probability with NADC30-like PRRSV *in vivo*

**Fig 7:**
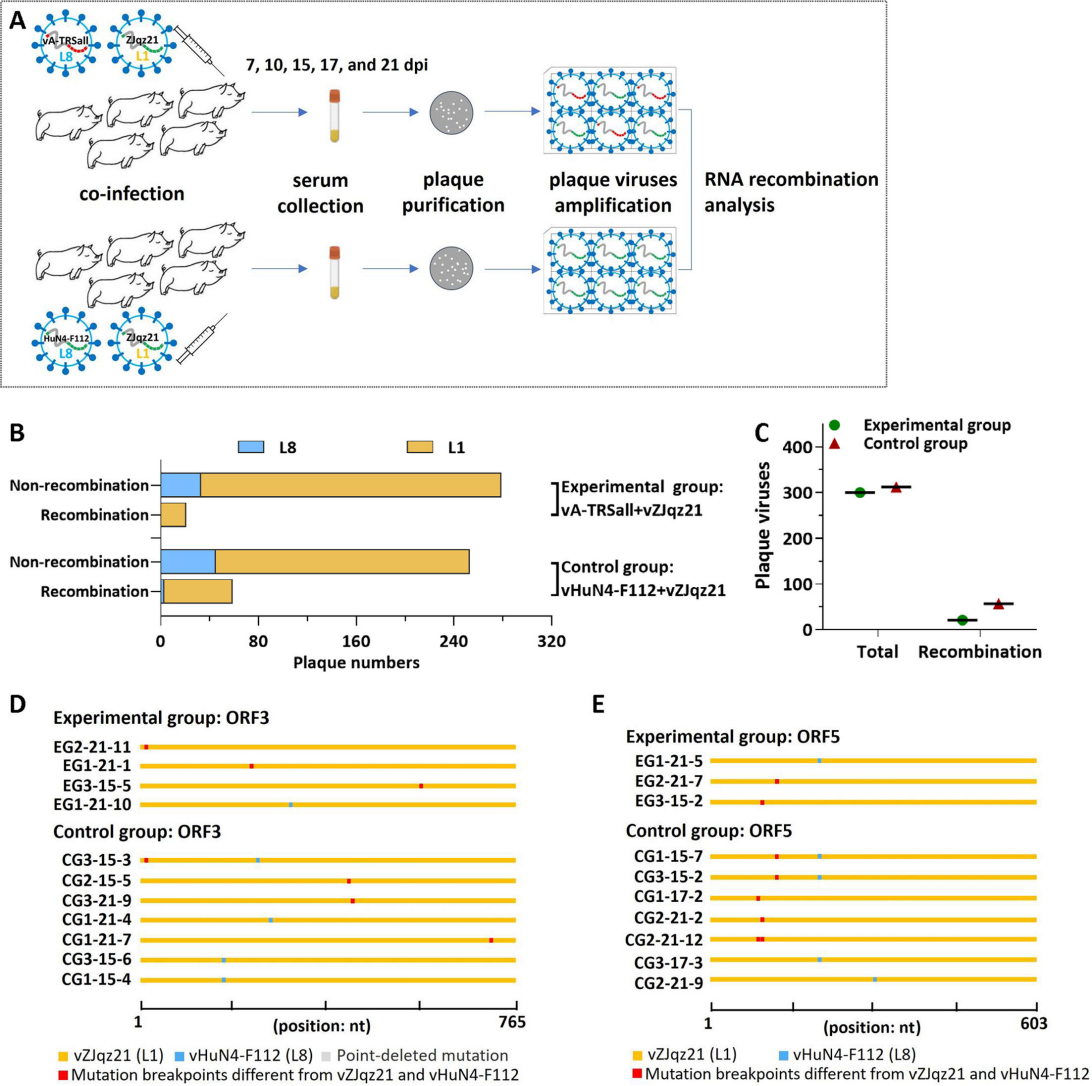
Recombination analysis of *in vivo* co-infection. (**A**) Schematic showing the *in vivo* recombination assay. Ten piglets were divided into two groups: the experimental group vA-TRSall co-infected with vZJqz21 and the control group vHuN4-F112 co-infected with vZJqz21. Serum samples were collected at 7, 10, 14, 17, and 21 dpv for plaque purification and sequencing to analyze the chance of recombination. (**B**) The proportion of recombinant PRRSVs in the plaque viruses from the experimental group (vA-TRSall and vZJqz21 co-infection) and control group (vHuN4-F112 and vZJqz21 co-infection). (**C and D**) A map of recombination patterns in ORF3 (**C**) or ORF5 (**D**) of the recombinant plaque viruses from the experimental or control groups. Each plaque virus is identified on the left, and the corresponding major parent is identified in different lineage colors on the right. A change in color indicates that the region has a recombinant.

Five 6-week-old piglets were co-infected with vA-TRSall and vZJqz21 (experimental group), and five piglets were co-infected with vHuN4-F112 and vZJqz21 (control group) simultaneously and monitored for 21 days (Fig. 7A) to evaluate the probability of RNA recombination *in vivo*. A total of 50 viruses were isolated from the serum samples of the two groups collected at 7, 10, 14, 17, and 21 days post-infection (dpi) and then used for plaque purification. Two hundred plaque viruses were isolated from the experimental and control groups, and whole-genome sequencing was conducted to analyze the proportion of recombinant viruses using SimPlot and RDP4 software. The amount of recombinant viruses in the experimental group was less than that in the control group (Fig. 7B). For most recombinants in the control group, the major parental strain was vZJqz21 (Fig. 7C), indicating that NADC30-like PRRSV provided the backbone for most recombination events. The recombination patterns of the recombinant plaque viruses from the experimental and control groups are displayed according to their specific positions in the gene region between ORF3. The mutation breakpoints for the ORF3 gene at the sites of 16, 167, and 689 nt showed the highest frequency (Fig. 7D; Table 2). The positions of the recombinant breakpoints were concentrated in the sites of 129 and 204 nt in the gene region between ORF5 (Fig. 7E; Table 2). Currently, all recombinants with a mutation breakpoint tend to use the NADC30-like PRRSV as the skeleton, resulting in a mutation breakpoint for HP-PRRSV (Fig. 7D and E). In the experimental group, the number of recombinants in the ORF3 and ORF5 genes was fewer than that in the control group. Compared with the control group, the forms of the mutation breakpoints produced by ORF3 and ORF5 significantly decreased in the experimental group (Table 2), further suggesting that the PRRS-attenuated vaccine strain with TRS circuit rewiring will significantly reduce the recombination probability with NADC30-like PRRSV *in vivo*.

**TABLE 2:** Putative recombination breakpoints upon co-infection *in vivo^a^^,^^b^*

Representative recombinant virus	Breakpoint number	Position	Number of recombinants
Experimental group (vA-TRSall and vZJqz21 co-infection)			
ORF5 gene			
EG1-21-a	1	204	4
EG2-21-b	1	129	2
EG3-15-c	1	99	8
ORF3 gene			
EG2-21-d	1	16	2
EG1-21-e	1	272	3
EG3-15-5	1	579	1
EG1-21-10	1	298	1
Control group (vHuN4-F112 and vZJqz21 co-infection)			
ORF5 gene			
CG1-15-f	2	129 and204	6
CG3-15-g	2	129 and 204	10
CG1-17-h	1	97	6
CG2-21-2	1	99	1
CG3-21-12	2	97 and 99	1
CG3-17-i	1	204	2
CG2-21-9	1	305	1
ORF3 gene			
CG3-15-j	2	16 and 276	2
CG2-15-k	1	445	4
CG3-21-9	1	447	1
CG1-21-l	1	298	2
CG1-21-m	1	689	5
CG3-15-n	1	167	9
CG1-15-o	1	167	7

^*a*^ X-Y-Z (X = serial number of piglets in the experimental or control groups, respectively; Y = dpi; Z = serial number of plaque viruses).

^*b*^ a: plaques EG1-21-(5,10-12); b: plaques EG2-21-(7,10); c: plaques EG3-15-(2-7,11-12); d: plaques EG2-21-(11,12); e: plaques EG1-21-(1-3); f: plaques CG1-15-(7-12); g: plaques CG3-15-(2-8, 10-12); h: plaques CG1-17-(2,5-7,9,11); i: plaques CG3-17-(3,6); j: plaques CG3-15-(3,5); k: plaques CG2-15-(1-3,5); l: plaques CG1-21-(4,8); m: plaques CG1-21-(1-3,5,7); n: plaques CG3-15-(3-11); o:plaques CG1-15-(4,6-11).

## DISCUSSION

Live-attenuated vaccines can provide highly effective protection against diseases and help to prevent the spread of pathogenic viruses. However, the development of safe and effective live-attenuated vaccines is currently a challenging trial-and-error process. In this study, the most common recombination patterns of the currently prevalent PRRSV strains were systematically analyzed (Fig. 1), and a rewiring strategy for the entire TRS circuit in the PRRSV genome was proposed to reduce the probability of RNA recombination. A series of TRSs mutations in the genome of the PRRSV live-attenuated vaccine strain HuN4-F112 were identified using reverse genetic manipulation. However, after analysis, only one recombinant mutant virus, vA-TRSall, with the whole TRS cassette mutation, was successfully rescued (Fig. 2 and 3). The immune efficacy against HP-PRRSV (vHuN4) and NADC30-like PRRSV (vZJqz21) was evaluated (Fig. 5 and 6), and *in vivo* and *in vitro* co-infection experiments with vZJqz21 were performed to evaluate its recombination resistance (Fig. 4 and 7). The mutant vaccine candidate was recombined with wild-type PRRSVs; however, this meant that the core nucleotide sequences of the TRS circuits were inconsistent, and the original base pairing was destroyed. The generated recombinant virus consequently lost its propagation activity, or its replication ability was hindered, and thus, a fatal genetic trap was introduced during the generation of RNA recombinant progeny virus. This kind of anti-recombinant vaccine could greatly reduce the probability of RNA recombination. The first outbreak of HP-PRRSV in China was in 2006, and it resulted in huge economic losses for the Chinese swine industry (23). In 2014, many PRRSV isolates with unique genetic backgrounds were reported in Southern China, and they were found to show their highest nucleotide identities with viruses reported in the US in 2008, represented by NADC30 (24). Two years after 2014, the NADC30-like PRRSV spread to many provinces in China and became a dominant epidemic strain in local vaccination pig farms (25). The NADC30-like strain belongs to PRRSV-2; however, compared to HP-PRRSV, its virulence is relatively low, and it mainly leads to respiratory symptoms in piglets, with a mortality of 30%–50% (26). Currently, the main method for preventing the spread of PRRSV in China is vaccination, and a live-attenuated vaccine is thus an important tool for disease control (27). In China, several vaccine strains (IngelvacPRRS, CH-1R, R98, JXA1-P80, TJM-F92, HuN4-F112, and GDr180) are currently widely used on pig farms (Fig. 1). In recent years, many new strains derived from the recombination of wild and vaccine strains have been reported (28, 29). The virulence of these recombinant viral strains is higher than that of the vaccine strain (30). RNA recombination plays a key role in the diversity and evolution of PRRSV, and numerous studies have confirmed the occurrence of recombination events in PRRSV, both *in vitro* and *in vivo* (31, 32). In recent years, with the introduction of L1 PRRSVs, including PRRSV-1, the number of PRRSV recombination events has increased in China (33, 34).

Generally, the recombination situation is distinguished based on the generated crossover junction: homologous recombination usually occurs at the same position of two parental templates, and the generated progeny recombinants have the same genetic structure as the parental templates, whereas nonhomologous recombination predominantly occurs at different sites, resulting in duplications, deletions, or insertions in the offspring genome. Homologous recombination usually occurs between related viruses with similar gene structures, whereas nonhomologous recombination can occur between two molecules of different origins. The potential recombination mechanism of the observed offspring recombinants remains unknown. However, in theory, different mechanisms can produce recombinants with similar structures, and consequently, the recombination mechanism should be considered separately according to the different recombinants (35). There are two generally accepted recombination mechanisms for RNA: the template-switching model and the breaking-reconnecting model (36). RdRp plays an important role in the template-switching mechanism, which can change the template during the synthesis of a new strand and produce a chimeric genome containing two parental template fragments. The model assumes that the nascent strand can be separated from the donor RNA molecule, combined with different receptor RNA molecules or at different positions in the donor RNA molecule, and RNA synthesis can continue; that is, homologous or nonhomologous recombinants can be produced (37). According to the internal relations between the replication template exchange mechanism and transcription regulatory sequence, the main purpose of this study was to disrupt the template-switching recombination between the mutant vaccine strain and the epidemic wild strains by rewiring the TRS circuit in the PRRSV genome. This concept can be found in the earlier research and utilization of the main mechanism of transcriptional regulatory sequences in the genome (38).

The PRRSV genome uses discontinuous transcription to synthesize standard sg mRNAs, which are conserved in arteritis viruses and coronaviruses (39, 40). During this process, PRRSV can produce chimeric mRNA molecules fused by an identical segment of the 5′ leader sequence and several non-adjacent regions of the 5′ genomes located in each ORF. However, TRS was found to be a combination of TRS-L-5′ and TRS-B-3′ sequences in the combination position of the chimeric mRNA (41). TRS contains a 6–7 nt conserved core sequence (CS) shared by TRS-L and TRS-B, which is crucial for the TRS cassette (42). Template-switching recombination is closely associated with TRS. During the synthesis of the new negative strand, a double strand is formed between CS-L and CS-B of the complementary strand, which leads to a switch of RdRp from the body sequence to the 5′-end leader sequence. If this complementary double strand does not occur, RdRp continues to synthesize the negative strand until it encounters the subsequent TRS-B in the positive strand of the genome, allowing the template to switch to either the leading strand of the 5′ positive strands or the complete anti-genomic strand (43). The stability of the CS in TRS is the key determinant of the occurrence and rate of template switching. Previous studies have shown that changing the conserved base of CS in TRS-L or TRS-B might lead to the exposure of new complementary sites in the virus genome, resulting in the generation of new small sgRNAs, which will destroy the transcription of sg mRNAs necessary for virus synthesis, leading to the inability of the virus to survive or weaken its replication ability (38, 44). According to a large number of data surveys, conservative base pairing in the CS of TRS-L and TRS-B in PRRSV genomes is in the “CC,”” which explains why template-switching recombination is common in the natural environment.

According to the above inference, the development of a live attenuated PRRSV vaccine by TRS circuit rewiring in this study is completely different from the TRS-L and TRS-B of PRRSV strains in the natural environment (Fig. 2). Transfection rescue results indicate that only the whole TRS circuit mutation produces infectious progeny virus with parental virus characteristics, confirming that the base pairing of TRS-L and TRS-B (TRS-2, TRS-3, TRS-4, TRS-5, TRS-6, and TRS-7) is essential for virus survival. This finding also demonstrated that the recombinant viruses with TRS circuit rewiring cannot survive, even if partial recovery mutations occur, further illustrating the genetic stability of vA-TRSall. After template-switching recombination with epidemic strains, TRS-L and TRS-B in the recombinant progeny viruses cannot complement each other, and consequently, they cannot survive, or their replication ability is weakened. By rewiring of the whole TRS circuit of the vHuN4-F112 vaccine strain, the full-length infectious clone of PRRSV with a rewired TRS circuit was constructed, rescued, and named vA-TRSall. The growth characteristics of the virus were similar to those of the parental virus vHuN4-F112 (Fig. 3). Immune efficacy experiments showed that vA-TRSall provided adequate immune protection against HP-PRRSV and vZJqz21, indicating that TRS circuit rewiring did not affect its homologous and heterogeneous protection capabilities (Fig. 5 and 6). To evaluate the recombination resistance capability of the vA-TRSall, *in vitro* co-infection tests were conducted using target cells and an *in vivo* co-infection test in piglets (Fig. 4 and 7). The results showed that compared with the vHuN4-F112 co-infection group, the vA-TRSall co-infection group reduced the detectable mutation breakpoints by approximately 10%, indicating that TRS circuit rewiring could reduce the probability of template-switching recombination with the epidemic strain. However, this study was a short-term co-infection monitoring test conducted under laboratory conditions. According to available research, most vaccine and epidemic strains recombine to produce new strains, which are monitored on farms that have been vaccinated regularly for a long time. The purpose of this study was to make a preliminary application attempt based on previous research on TRS and template-switching mechanisms (38) to develop an anti-recombinant vaccine of PRRSV by rewiring the TRS circuit to effectively reduce the probability of RNA recombination between vaccine strains and epidemic wild strains, as well as the application of PRRSV TRS in our lab.

## MATERIALS AND METHODS

### Recombination analysis

Nine representative strains were used as the reference parents: NADC30 (L1), XW008 (L2), QYYZ (L3), EDRD-1 (L4), VR-2332 (L5), P129 (L6), SP (L7), JXA1 (L8), and MN30100 (L9). Interlineage recombination events were preliminarily detected using SimPlot software, and recombinant parental strains and breakpoint locations were determined. A recombination detection program (RDP4 software) was used to analyze the identified recombinant strains further.

### Cells and viruses

MARC-145 cells (ATCC, Manassas, VA, USA) and PAMs were cultured as described previously (45). The HP-PRRSV strain vHuN4 (accession number: EF635006), HP-PRRSV-attenuated vaccine strain vHuN4-F112, and the NADC30-like strain vZJqz21 (accession number: OK274266) were isolated and preserved in our laboratory (22).

### Construction and rescue of mutant viruses containing redesigned TRS circuits

Based on the possibility of a “G_5_G_6_” mutation of “C_5_C_6_” in the core six nucleotides of the TRS cassette, which does not affect the coding of genomic amino acids, the core six nucleotides of all TRSs, including the leader TRS in pHuN4-F112, were substituted into “G_5_G_6_.” At the same time, to simulate the possible progeny viruses of the PRRSV strain with a TRS circuit mutation that recombines with an epidemic strain by template switching in the natural environment, six possible progeny recombinants were constructed, referred to as the TRS cassette chimeric mutant series. Virus rescue tests were then conducted to verify whether the mutations could cause lethal effects or seriously affect the virus replication ability. The recombinant TRS cassette mutant plasmids were pHuN4TRSall, pHuN4TRSL, pHuN4TRS-LB2-6, pHuN4TRS-LB7, pHuN4TRS-B2-7, pHuN4TRS-B7, and pHuN4TRS-B2-6. Using the six pairs of amplification primers listed in Table S3, the whole genome was amplified by segmented PCR with Q5 high-fidelity enzyme (Thermo Fisher Scientific, Waltham, MA, USA), and the PCR products were ligated with the TA/Blunt-Zero cloning vector (Vazyme, Nanjing, China). Site-directed mutagenesis was performed on the leading TRS and body TRSs fragments, and after restriction endonuclease digestion, the whole genome was connected to the vector pBluescript II SK (+), according to the genome sequence. The enzymatic cleavage sites used in this study were *Pac* I, *Mfe* I, *BamH* I, *Cla* I, *Asc* I, *Mlu* I, and *Swa* I (New England BioLabs, Ipswich, MA, USA). Sequential fragments were used to construct full-length infectious clones of the PRRSV genome. The *Swa* I enzyme digestion site was used to linearize all recombinant PRRSV full-length infectious clone plasmids, which were purified by agarose gel electrophoresis and used as the templates for T7 *in vitro* transcription. The RNA transcribed *in vitro* was transfected into BHK-21 cells using the DMRIE-C transfection reagent. After 72 h, the supernatant of the BHK-21 cells was collected and passaged continuously onto MARC-145 cells. CPEs were observed every day, and the virus rescue test was repeated three times to assess virus rescue and determine whether the mutation would make the virus lethal or affect the level of replication. RNA secondary structure analysis was performed as previously described (46).

### Viral characteristics of the mutant virus with TRS circuit rewiring

The rescued mutant virus was successively passed on MARC-145 cells to passage 5 (P5), and the viral titers were assessed using the Reed-Muench method (47). The P5 generation vA-TRSall and parental virus vHuN4-F112 were inoculated into MARC-145 cells at a density of 80%, and the viral supernatants were discarded 48 h after infection. For WB, cells were washed twice and lysed with lysis buffer. Protein samples were prepared and resolved on 10% SDS polyacrylamide gels and transferred. Membranes were blocked with 5% skim milk for 2 h at room temperature and incubated overnight at 4°C with the primary antibodies. The anti-N protein polyclonal antibody and anti-nsp10 monoclonal antibody were produced in our lab. The anti-β-actin antibody was purchased from Sigma (A5441, Sigma-Aldrich, St. Louis, MO, USA). After washing with Tris-buffered saline with 0.1% Tween-20, blots were incubated with horseradish peroxidase-conjugated goat anti-mouse secondary antibody (SA00001-1, Proteintech Group, Inc., Chicago, USA) for 1 h at room temperature and developed using Pierce ECL substrate (Thermo). For IFA, cells were fixed with low-temperature 80% ethanol, and PRRSV N protein and nsp10 antibody were diluted with 1% bovine serum albumin (BSA) at a dilution ratio of 1:1,000 and incubated in a 37°C incubator for 1 h. Goat anti-mouse IgG (H + L), highly cross-adsorbed, was used as the secondary antibody (A-11029/A-11004, Invitrogen, Thermo) with 1% BSA at a dilution ratio of 1:1,000, and green fluorescent secondary antibody at a dilution ratio of 1:800 was incubated for 45 min and observed under a fluorescence inverted microscope. For plaque morphology assay, MARC-145 cells in six-well plates were infected with the mutant or parental viruses (MOI = 0.01). After incubating at 37°C for 1 h, the virus diluent was discarded, 2% low melting point agarose (Promega, Madison, WI, USA), 2 × MEM (Gibico, Thermo), and 4% fetal bovine serum (FBS; Gibico, Thermo) were added and placed into a six-well plate. After inoculating at room temperature, the plate was inverted in an incubator at 37°C for 4 dpi. After the viral plaque appeared, 2 mL of 4% formaldehyde solution was added to the well, the gel was removed, and the shape and number of plaques were observed by staining with a 5% crystal violet solution. The growth kinetics of the MARC-145 cells and PAMs were studied by infecting them with mutant or parental viruses, respectively (MOI = 0.01). After 1 h of virus adsorption, the cells were washed and incubated with 2% FBS in DMEM (Gibico, Thermo) at 37°C. The viral infection supernatants were collected at different time points, the virus titer was measured, and a multistep growth curve of the mutant and parental viruses was drawn. Analysis of (−) gRNAs and sg mRNAs was performed as previously described (48). Northern blotting was performed as previously described (46).

### Immune efficacy evaluation of the mutant virus

Fifteen 6-week-old landrace PRRSV-, African swine fever virus (ASFV)-, and classical swine fever virus (CSFV)-free piglets were screened. They were randomly divided into three groups with five piglets in each group, namely, the vA-TRSall, BC, and mock groups, as shown in Fig. S1. Each treatment group was administered individually. The immunization group was inoculated by intramuscular injection into the neck, and each pig was inoculated with 10^5.5^ TCID_50_ vA-TRSall or DMEM 2 mL. On day 28 after immunization, 2 mL of the HP-PRRSV strain vHuN4 containing 10^4^ TCID_50_ was inoculated into each pig in vA-TRSall group and BC group, and 2 mL of DMEM was inoculated into each pig in the mock group. Piglets were monitored daily to assess their overall health and rectal temperature. An evaluation of clinical signs in each of the three groups was performed as previously described (22, 49). Serum samples were collected from piglets at 5, 7, 10, 14, 21, 28, 35, 42, and 49 days of age for detection. All surviving animals were euthanized and autopsied on day 49. A professional veterinary pathologist guided and performed the animal experiments. The second batch of experiments was conducted under the same conditions as described above. Fifteen piglets were randomly divided into three groups, named the vA-TRSall, BC2, and mock2 groups, respectively, as shown in Fig. S2. The challenge virus was the NADC30-like strain vZJqz21 (10^5^ TCID_50_), which was inoculated into each pig in vA-TRSall group and BC group, and 2 mL of DMEM was inoculated into each pig in the mock group.

### *In vitro* co-infection recombination analysis

The mutant virus vA-TRSall or the parental virus vHuN4-F1112 were used to co-infect PAMs with the epidemic NADC30-like strain vZJqz21. The experimental group was co-infected with vA-TRSall and vZJqz21 (MOI = 1), and the control group was co-infected with vHuN4-F112 and vZJqz21 (MOI = 1). Viral supernatants were collected at 12, 24, 36, and 48 h after co-infection, and the viral load at different time points was detected using RT-qPCR. The viral supernatant collected at the time point with the highest viral load was selected for the plaque purification test. The viral supernatants were diluted by times at the ratio of 1:10, and 300 µL of virus diluted 10^1^–10^6^ times was used to infect MARC-145 cells in six-well plates. After incubating at 37°C for 1 h, the virus diluent was discarded, 2% low melting point agarose (Promega), 2 × MEM (Gibico, Thermo), and 4% FBS (Gibico, Thermo) were added and placed into a six-well plate. After inoculating at room temperature, the plate was inverted in an incubator at 37°C for 4–6 dpi. After the viral plaques appeared, 40 plaques were selected and passaged continuously onto fresh MARC-145 cells to obtain the plaque viruses. The whole genomes of all plaque viruses were sequenced and compared with that of the parental virus. Plaque viruses were named as X-Y-Z (X = EG or CG; representing the experimental or control groups, respectively; Y = hpi, Z = serial number of plaque viruses).

### *In vivo* co-infection recombination analysis

Ten 6-week-old piglets were selected that tested negative for PRRSV, CSFV, and ASFV antibodies (IDEXX, Westbrook, MA, USA). They were divided into two groups: an experimental group (vA-TRSall co-infected with vZJqz21) and a control group (vHuN4-F112 co-infected with vZJqz21). The total amount of virus inoculated was 2 mL per piglet, and each mL contained 10^5^ TCID_50_ of the virus. Serum samples were collected 7, 10, 15, 17, and 21 days after inoculation, and the viruses were isolated from the serum samples. Each isolated virus was purified from plaques. Two hundred plaques were selected from each group. The genomes of all plaque viruses were sequenced and compared with that of the parental virus. The plaque viruses were named as X-Y-Z (X = serial number of piglets in the experimental or control groups, respectively; Y = dpi; Z = serial number of plaque viruses).

### Statistical analysis

All experiments were performed at least three times independently, and statistical significance was analyzed using *t* tests. *P* < 0.05 was considered statistically significant.

## Data Availability

All study data are included in the article and supplemental material.

## SUPPLEMENTAL MATERIAL

Supplemental material mbio.02350-24-s0001.pdf Figures S1 and S2; Tables S1 to S3.

ASM does not own the copyrights to Supplemental Material that may be linked to, or accessed through, an article. The authors have granted ASM a non-exclusive, world-wide license to publish the Supplemental Material files. Please contact the corresponding author directly for reuse.

## References

[1] Liu D, Zhou R, Zhang J, Zhou L, Jiang Q, Guo X, Ge X, Yang H. 2011. Recombination analyses between two strains of porcine reproductive and respiratory syndrome virus in vivo. Virus Res 155:473–486. doi:10.1016/j.virusres.2010.12.003 21167230

[2] Muslin C, Mac Kain A, Bessaud M, Blondel B, Delpeyroux F. 2019. Recombination in enteroviruses, a multi-step modular evolutionary process. Viruses 11:859. doi:10.3390/v11090859 31540135 PMC6784155

[3] Nagy PD, Bujarski JJ. 1997. Engineering of homologous recombination hotspots with AU-rich sequences in brome mosaic virus. J Virol 71:3799–3810. doi:10.1128/JVI.71.5.3799-3810.1997 9094655 PMC191530

[4] Lai MM. 1992. RNA recombination in animal and plant viruses. Microbiol Rev 56:61–79. doi:10.1128/mr.56.1.61-79.1992 1579113 PMC372854

[5] Renukaradhya GJ, Meng XJ, Calvert JG, Roof M, Lager KM. 2015. Live porcine reproductive and respiratory syndrome virus vaccines: current status and future direction. Vaccine (Auckl) 33:4069–4080. doi:10.1016/j.vaccine.2015.06.092 26148878

[6] Sun Q, Xu H, An T, Cai X, Tian Z, Zhang H. 2023. Recent progress in studies of porcine reproductive and respiratory syndrome virus 1 in China. Viruses 15:1528. doi:10.3390/v15071528 37515213 PMC10384046

[7] Shi M, Lam T-Y, Hon C-C, Murtaugh MP, Davies PR, Hui R-H, Li J, Wong L-W, Yip C-W, Jiang J-W, Leung F-C. 2010. Phylogeny-based evolutionary, demographical, and geographical dissection of North American type 2 porcine reproductive and respiratory syndrome viruses. J Virol 84:8700–8711. doi:10.1128/JVI.02551-09 20554771 PMC2919017

[8] Li Y, Jiao D, Jing Y, He Y, Han W, Li Z, Ma Z, Feng Y, Xiao S. 2022. Genetic characterization and pathogenicity of a novel recombinant PRRSV from lineage 1, 8 and 3 in China failed to infect MARC-145 cells. Microb Pathog 165:105469. doi:10.1016/j.micpath.2022.105469 35271985

[9] Cui X-Y, Xia D-S, Huang X-Y, Tian X-X, Wang T, Yang Y-B, Wang G, Wang H-W, Sun Y, Xiao Y-H, Tian Z-J, Cai X-H, An T-Q. 2022. Recombinant characteristics, pathogenicity, and viral shedding of a novel PRRSV variant derived from twice inter-lineage recombination. Vet Microbiol 271:109476. doi:10.1016/j.vetmic.2022.109476 35679815

[10] Trevisan G, Magstadt D, Woods A, Sparks J, Zeller M, Li G, Krueger KM, Saxena A, Zhang J, Gauger PC. 2023. A recombinant porcine reproductive and respiratory syndrome virus type 2 field strain derived from two PRRSV-2-modified live virus vaccines. Front Vet Sci 10:1149293. doi:10.3389/fvets.2023.1149293 37056231 PMC10086154

[11] Xia Y, Zhang T, Gong D, Qi J, Jiang S, Yang H, Zhu X, Gan Y, Zhang Y, Han Y, Li Y, Li J. 2023. Recombination and mutation in a new HP-PRRSV strain (SD2020) from China. Viruses 15:165. doi:10.3390/v15010165 36680205 PMC9864264

[12] Pan J, Zeng M, Zhao M, Huang L. 2023. Research progress on the detection methods of porcine reproductive and respiratory syndrome virus. Front Microbiol 14:1097905. doi:10.3389/fmicb.2023.1097905 36970703 PMC10033578

[13] Kong C, Li D, Hu Y, Gao P, Zhang Y, Zhou L, Ge X, Guo X, Han J, Yang H. 2023. The genetic variation of porcine reproductive and respiratory syndrome virus replicase protein nsp2 modulates viral virulence and persistence. J Virol 97:e0168922. doi:10.1128/jvi.01689-22 36916907 PMC10062138

[14] Fang Y, Snijder EJ. 2010. The PRRSV replicase: exploring the multifunctionality of an intriguing set of nonstructural proteins. Virus Res 154:61–76. doi:10.1016/j.virusres.2010.07.030 20696193 PMC7114499

[15] Sha H, Zhang H, Chen Y, Huang L, Zhao M, Wang N. 2022. Research progress on the NSP9 protein of porcine reproductive and respiratory syndrome virus. Front Vet Sci 9:872205. doi:10.3389/fvets.2022.872205 35898550 PMC9309524

[16] Li Y, Zhou L, Zhang J, Ge X, Zhou R, Zheng H, Geng G, Guo X, Yang H. 2014. Nsp9 and Nsp10 contribute to the fatal virulence of highly pathogenic porcine reproductive and respiratory syndrome virus emerging in China. PLoS Pathog 10:e1004216. doi:10.1371/journal.ppat.1004216 24992286 PMC4081738

[17] Zhao K, Gao JC, Xiong JY, Guo JC, Yang YB, Jiang CG, Tang YD, Tian ZJ, Cai XH, Tong GZ, An TQ. 2018. Two residues in NSP9 contribute to the enhanced replication and pathogenicity of highly pathogenic porcine reproductive and respiratory syndrome virus. J Virol 92:e02209-17. doi:10.1128/JVI.02209-17 29321316 PMC5972891

[18] Music N, Gagnon CA. 2010. The role of porcine reproductive and respiratory syndrome (PRRS) virus structural and non-structural proteins in virus pathogenesis. Anim Health Res Rev 11:135–163. doi:10.1017/S1466252310000034 20388230

[19] Smits SL, van Vliet ALW, Segeren K, el Azzouzi H, van Essen M, de Groot RJ. 2005. Torovirus non-discontinuous transcription: mutational analysis of a subgenomic mRNA promoter. J Virol 79:8275–8281. doi:10.1128/JVI.79.13.8275-8281.2005 15956573 PMC1143767

[20] Mori A, Lavezzari D, Pomari E, Deiana M, Piubelli C, Capobianchi MR, Castilletti C. 2023. sgRNAs: a SARS-CoV-2 emerging issue. Asp Mol Med 1:100008. doi:10.1016/j.amolm.2023.100008 37519862 PMC10105645

[21] Pasternak AO, Spaan WJM, Snijder EJ. 2006. Nidovirus transcription: how to make sense...? J Gen Virol 87:1403–1421. doi:10.1099/vir.0.81611-0 16690906

[22] Li L, Chen J, Cao Z, Cao Y, Guo Z, Tong W, Zhou Y, Li G, Jiang Y, Liu C, Yu L, Qiao S, Liu J, Tong G, Gao F. 2021. Recombinant bivalent live vectored vaccine against classical swine fever and HP-PRRS revealed adequate heterogeneous protection against NADC30-like strain. Front Microbiol 12:822749. doi:10.3389/fmicb.2021.822749 35069517 PMC8767063

[23] Tian K, Yu X, Zhao T, Feng Y, Cao Z, Wang C, Hu Y, Chen X, Hu D, Tian X, et al.. 2007. Emergence of fatal PRRSV variants: unparalleled outbreaks of atypical PRRS in China and molecular dissection of the unique hallmark. PLoS One 2:e526. doi:10.1371/journal.pone.0000526 17565379 PMC1885284

[24] Yu L, Zhao P, Dong J, Liu Y, Zhang L, Liang P, Wang L, Song C. 2017. Genetic characterization of 11 porcine reproductive and respiratory syndrome virus isolates in South China from 2014 to 2015. Virol J 14:139. doi:10.1186/s12985-017-0807-4 28738888 PMC5525233

[25] Dortmans J, Buter GJ, Dijkman R, Houben M, Duinhof TF. 2019. Molecular characterization of type 1 porcine reproductive and respiratory syndrome viruses (PRRSV) isolated in the Netherlands from 2014 to 2016. PLoS One 14:e0218481. doi:10.1371/journal.pone.0218481 31246977 PMC6597066

[26] Sui X, Guo X, Jia H, Wang X, Lin W, Li M, Gao X, Wu J, Jiang Y, Willems L, Zhu H, Xin T, Hou S. 2018. Genomic sequence and virulence of a novel NADC30-like porcine reproductive and respiratory syndrome virus isolate from the Hebei province of China. Microb Pathog 125:349–360. doi:10.1016/j.micpath.2018.08.048 30149129

[27] Nan Y, Wu C, Gu G, Sun W, Zhang Y-J, Zhou E-M. 2017. Improved vaccine against PRRSV: current progress and future perspective. Front Microbiol 8:1635. doi:10.3389/fmicb.2017.01635 28894443 PMC5581347

[28] Liu J, Wei C, Lin Z, Fan J, Xia W, Dai A, Yang X. 2019. Recombination in lineage 1, 3, 5 and 8 of porcine reproductive and respiratory syndrome viruses in China. Infect Genet Evol 68:119–126. doi:10.1016/j.meegid.2018.12.006 30529558

[29] Yu F, Yan Y, Shi M, Liu H-Z, Zhang H-L, Yang Y-B, Huang X-Y, Gauger PC, Zhang J, Zhang Y-H, Tong G-Z, Tian Z-J, Chen J-J, Cai X-H, Liu D, Li G, An T-Q. 2020. Phylogenetics, genomic recombination, and NSP2 polymorphic patterns of porcine reproductive and respiratory syndrome virus in China and the United States in 2014-2018. J Virol 94:e01813-19. doi:10.1128/JVI.01813-19 31896589 PMC7158704

[30] Zhou L, Wang Z, Ding Y, Ge X, Guo X, Yang H. 2015. NADC30-like strain of porcine reproductive and respiratory syndrome virus, China. Emerg Infect Dis 21:2256–2257. doi:10.3201/eid2112.150360 26584305 PMC4672414

[31] Franzo G, Cecchinato M, Martini M, Ceglie L, Gigli A, Drigo M. 2014. Observation of high recombination occurrence of porcine reproductive and respiratory syndrome virus in field condition. Virus Res 194:159–166. doi:10.1016/j.virusres.2014.08.005 25150757 PMC7127771

[32] Zhang X, Li Y, Xiao S, Yang X, Chen X, Wu P, Song J, Ma Z, Cai Z, Jiang M, Zhang Y, Yang Y, Zhang Z, Zhou Z, Sheng J, Wang H. 2019. High-frequency mutation and recombination are responsible for the emergence of novel porcine reproductive and respiratory syndrome virus in northwest China. Arch Virol 164:2725–2733. doi:10.1007/s00705-019-04373-z 31468140

[33] Li C, Li S, Li S, Qiu M, Lin H, Sun Z, Qiu Y, Qi W, Feng B, Li J, Zheng W, Yu X, Tian K, Shang S, Fan K, Zhu J, Chen N. 2023. Efficacy of a porcine reproductive and respiratory syndrome virus 1 (PRRSV-1) natural recombinant against a heterologous PRRSV-1 isolate both clustered within the subgroup of BJEU06-1-like isolates. Vet Microbiol 285:109847. doi:10.1016/j.vetmic.2023.109847 37625255

[34] Luo Q, Zheng Y, He Y, Li G, Zhang H, Sha H, Zhang Z, Huang L, Zhao M. 2023. Genetic variation and recombination analysis of the GP5 (GP5a) gene of PRRSV-2 strains in China from 1996 to 2022. Front Microbiol 14:1238766. doi:10.3389/fmicb.2023.1238766 37675419 PMC10477998

[35] Galli A, Bukh J. 2014. Comparative analysis of the molecular mechanisms of recombination in hepatitis C virus. Trends Microbiol 22:354–364. doi:10.1016/j.tim.2014.02.005 24636243

[36] Bentley K, Evans DJ. 2018. Mechanisms and consequences of positive-strand RNA virus recombination. J Gen Virol 99:1345–1356. doi:10.1099/jgv.0.001142 30156526

[37] Kim H, Ellis VD, Woodman A, Zhao Y, Arnold JJ, Cameron CE. 2019. RNA-dependent RNA polymerase speed and fidelity are not the only determinants of the mechanism or efficiency of recombination. Genes (Basel) 10:968. doi:10.3390/genes10120968 31775299 PMC6947342

[38] Yount B, Roberts RS, Lindesmith L, Baric RS. 2006. Rewiring the severe acute respiratory syndrome coronavirus (SARS-CoV) transcription circuit: engineering a recombination-resistant genome. Proc Natl Acad Sci U S A 103:12546–12551. doi:10.1073/pnas.0605438103 16891412 PMC1531645

[39] van Vliet ALW, Smits SL, Rottier PJM, de Groot RJ. 2002. Discontinuous and non-discontinuous subgenomic RNA transcription in a nidovirus. EMBO J 21:6571–6580. doi:10.1093/emboj/cdf635 12456663 PMC136939

[40] Zúñiga S, Sola I, Alonso S, Enjuanes L. 2004. Sequence motifs involved in the regulation of discontinuous coronavirus subgenomic RNA synthesis. J Virol 78:980–994. doi:10.1128/jvi.78.2.980-994.2004 14694129 PMC368802

[41] Sola I, Almazán F, Zúñiga S, Enjuanes L. 2015. Continuous and discontinuous RNA synthesis in coronaviruses. Annu Rev Virol 2:265–288. doi:10.1146/annurev-virology-100114-055218 26958916 PMC6025776

[42] Alonso S, Izeta A, Sola I, Enjuanes L. 2002. Transcription regulatory sequences and mRNA expression levels in the coronavirus transmissible gastroenteritis virus. J Virol 76:1293–1308. doi:10.1128/jvi.76.3.1293-1308.2002 11773405 PMC135778

[43] Menéndez-Arias L, Sebastián-Martín A, Álvarez M. 2017. Viral reverse transcriptases. Virus Res 234:153–176. doi:10.1016/j.virusres.2016.12.019 28043823

[44] Pasternak AO, van den Born E, Spaan WJM, Snijder EJ. 2003. The stability of the duplex between sense and antisense transcription-regulating sequences is a crucial factor in arterivirus subgenomic mRNA synthesis. J Virol 77:1175–1183. doi:10.1128/jvi.77.2.1175-1183.2003 12502834 PMC140805

[45] Li L, Wei Z, Zhou Y, Gao F, Jiang Y, Yu L, Zheng H, Tong W, Yang S, Zheng H, Shan T, Liu F, Xia T, Tong G. 2015. Host miR-26a suppresses replication of porcine reproductive and respiratory syndrome virus by upregulating type I interferons. Virus Res 195:86–94. doi:10.1016/j.virusres.2014.08.012 25218480 PMC7114497

[46] Gao F, Lu J, Yao H, Wei Z, Yang Q, Yuan S. 2012. Cis-acting structural element in 5' UTR is essential for infectivity of porcine reproductive and respiratory syndrome virus. Virus Res 163:108–119. doi:10.1016/j.virusres.2011.08.018 21924304 PMC7114472

[47] Thakur AK, Fezio WL. 1981. A computer program for estimating LD50 and its confidence limits using modified Behrens-Reed-Muench cumulant method. Drug Chem Toxicol 4:297–305. doi:10.3109/01480548109018136 7338208

[48] Gao F, Yao H, Lu J, Wei Z, Zheng H, Zhuang J, Tong G, Yuan S. 2013. Replacement of the heterologous 5' untranslated region allows preservation of the fully functional activities of type 2 porcine reproductive and respiratory syndrome virus. Virology (Auckl) 439:1–12. doi:10.1016/j.virol.2012.12.013 PMC7111940 23453581

[49] Jiang Y, Xia T, Zhou Y, Yu L, Yang S, Huang Q, Li L, Gao F, Qu Z, Tong W, Tong G. 2015. Characterization of three porcine reproductive and respiratory syndrome virus isolates from a single swine farm bearing strong homology to a vaccine strain. Vet Microbiol 179:242–249. doi:10.1016/j.vetmic.2015.06.015 26162970

