# Supplementary material for: Engineering a live-attenuated porcine reproductive and respiratory syndrome virus vaccine to prevent RNA recombination by rewiring transcriptional regulatory sequences
Source: mBio. 2024 Dec 23;16(2):e02350-24. doi: 10.1128/mbio.02350-24 (PMC11796407; doi:10.1128/mbio.02350-24)
Supplement: Supplemental material — Figures S1 and S2; Tables S1 to S3. [file mbio.02350-24-s0001.pdf]

Supporting Information for

Engineering a live-attenuated Porcine Reproductive and Respiratory Syndrome Virus vaccine to prevent RNA recombination by rewiring transcriptional regulatory sequences

Liwei Li, Jinxia Chen, Zhengda Cao, Ziqiang Guo, Jiachen Liu, Yanjun Zhou, Guangzhi Tong, Fei Gao

Corresponding author: Fei Gao

Email: feigao@shvri.ac.cn

This PDF file includes:

Figures S1 to S2

Tables S1 to S3

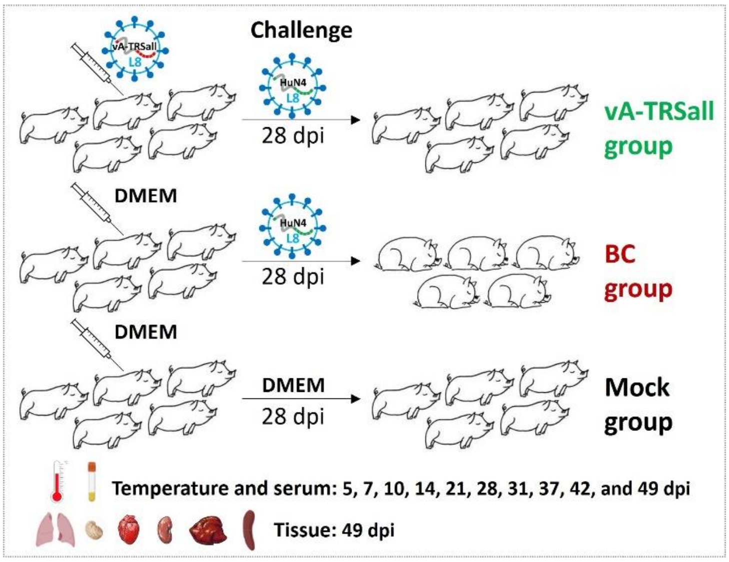

FIG S1 Schematic diagram of immune efficacy evaluation of vA-TRSall against HP-PRRSV HuN4 strain.

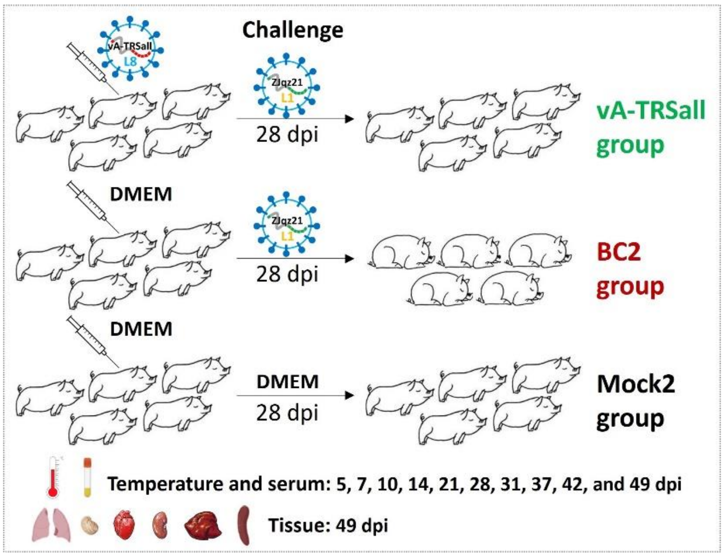

FIG S2 Schematic diagram of immune efficacy evaluation of vA-TRSall against NADC30-like ZJqz21 strain.

Table S1 Information of PRRSV-2 in China.

Accession number	Strain	Province	Date	Lineage
OR766560.1	SCABTC-202309	Sichuan	2023	L1
OR575928.1	HN-NY/2023	Henan	2023	L1
OR711915.1	GD-7	Guangdong	2023	L1
OR662185.1	BDSP-1	Heibei	2023	L1
OR582383.1	GX-3	Guangdong	2023	L1
OR826316.1	TZJ3116	Heilongjiang	2023	L1
OR826315.1	TZJ3115	Heilongjiang	2023	L1
OR826314.1	WK730	Heilongjiang	2023	L1
OR826313.1	TZJ3005	Heilongjiang	2023	L1
OR269980.1	2023GD-4	Jiangsu	2023	L1
OR753369.1	CHNMGKL1-2304	Inner Mongolia	2023	L1
OR365672.1	SCABTC-202305	Sichuan	2023	L1
OR365675.1	SCABTC-202308	Sichuan	2023	L1
OR365673.1	SCABTC-202306	Sichuan	2023	L5
OR365674.1	SCABTC-202307	Sichuan	2023	L5
OR670493.1	SCCD22	Sichuan	2022	L1
OR800933.1	hy_2203	Guangdong	2022	L1
OQ538073.1	SDQD95	Heilongjiang	2022	L8
OQ538074.1	SDYT91	Heilongjiang	2022	L8
OR369723.1	GZ2022	Hubei	2022	L1
OR146747.1	SF5	Hubei	2022	L1
OR146748.1	SF7	Hubei	2022	L1
OR146749.1	ZH12	Hubei	2022	L8
OP716076.1	CH-HNPY-01/2022	Henan	2022	L1
ON142049.1	PRRSV-HQ-2020	Zhejiang	2020/2022	L8
OR800932.1	sg_2107	Guangdong	2021	L1
OR800931.1	zq_2109	Guangdong	2021	L8
OR800930.1	qy_2104	Guangdong	2021	L1
OR800929.1	fs_2108	Guangdong	2021	L8
OR800927.1	sg_2108	Guangdong	2021	L8
OR800925.1	qy_2105	Guangdong	2021	L1
OR800926.1	sg_2104	Guangdong	2021	L8
OR800924.1	zq_2108	Guangdong	2021	L8
OR468246.1	GXNN20210906	Guangxi	2021	L3
OR247780.1	XJSW-2021	Xinjiang	2021	L8
OR102499.1	ZH2021	Hubei	2021	L8
OQ817851.1	PRRSV/TZJ1712	Jiangsu	2021	L8
OQ817852.1	PRRSV/SD-2021-3-10	Shandong	2021	L8
OQ871558.1	HN-1	Hubei	2021	L1
OR800928.1	qy_2008	Guangdong	2020	L8
OR518274.1	SXht2012	Shanxi	2020	L1
OR102498.1	SH	Hubei	2020	L1
OR066233.1	HuBXW	Hubei	2020	L1
OQ817849.1	PRRSV/DY	Heilongjiang	2020	L8
OQ817850.1	PRRSV/WK357	Jiangsu	2020	L5
OR102497.1	FJ	Hubei	2019	L8
OR102496.1	XJ	Hubei	2018	L8
OQ817848.1	PRRSV/NA80-lun	Heilongjiang	2018	L5
OQ924467.1	HM1801	Beijing	2018	L8
OQ924468.1	HM1805	Beijing	2018	L1
OQ924469.1	HM1807	Beijing	2018	L8
OQ924470.1	HM1809	Beijing	2018	L1
OQ924471.1	HM1810	Beijing	2018	L8
OQ924472.1	HM1811	Beijing	2018	L8
OQ924473.1	HM1812	Beijing	2018	L8
OQ924466.1	HM1710	Beijing	2017	L8
OR250810.1	HuN-ZZ	Guangxi	2022	L8
OR115682.1	BZ-4-19	Hebei	2023	L5
OQ986592.1	SCABTC-202303	Sichuan	2023	L8
OQ986591.1	SCABTC-202302	Sichuan	2023	L1
OQ986590.1	SCABTC-202301	Sichuan	2023	L8
OQ986589.1	SCABTC-202304	Sichuan	2023	L8
OQ944349.1	YN-DZ	Yunan	2023	L8
OQ883907.1	SCSN2020	Sichuan	2020	L8
OQ817853.1	XJ-1	Xinjiang	2021	L1
OQ790147.1	WK621	Heilongjiang	2022	L1
OQ790146.1	TZJ2451	Heilongjiang	2022	L1
OQ748875.1	TZJ2756	Heilongjiang	2022	L1
OQ735301.1	BL2019	Guangdong	2019	L1
OQ606399.1	GD2022	Guangdong	2022	L1
OQ506516.1	SDWH86	Shandong	2022	L8

OQ459668.1	GD20220303	Guangdong	2022	L8
OQ459667.1	GXYN20220502	Guangxi	2022	L8
OQ459666.1	GXYL20220501	Guangxi	2022	L1
OQ459665.1	GXHX20211106	Guangxi	2021	L1
OQ459664.1	GXGL20220301	Guangxi	2022	L1
OQ459663.1	GXGG20210301	Guangxi	2021	L8
OQ459662.1	GXBY20220301	Guangxi	2022	L1
OQ357725.1	CN/G8/2018	Fujian	2018	L1
OQ357724.1	FJLX06/2021	Fujian	2021	L8
OQ204111	GX20210501	Guangxi	2021	L1
OP852569.1	SH2020	Shanghai	2020	L8
OP805381.1	SDLY27-2022	Shandong	2022	L8
OP784966.1	HuN-ZJJ-A	Hunan	2021	L8
OP784965.1	HuN-SY-B	Hunan	2021	L8
OP784964.1	HuN-ZZ-B	Hunan	2021	L8
OP784963.1	HuN-XT-B	Hunan	2021	L1
OP764591.1	GSWW/2018	Gansu	2018/2022	L8
OP734318.1	CHbj2103	Beijing	2021/2022	L1
OP734317.1	CHbj2102	Beijing	2021/2022	L1
OP734316.1	CHbj2101	Beijing	2021/2022	L1
OP168793.1	SDHY_DZ037	Shandong	2020	L1
OP131596.1	HBag-4	Hebei	2020/2022	L1
ON691480	GD-F1	Guangdong	2021	L1
ON691479	GD-H1	Guangdong	2021	L1
ON584463	NA107-844/TW-2018	Taiwan	2018	L1
ON462051	GDXNF229-1811	Guangdong	2018	L3
ON462050	GXXNF78-1806	Guangxi	2018	L3
ON462049	GXXNF74-1806	Guangxi	2018	L3
ON462048	GDXNF60-1805	Guangdong	2018	L3
ON462047	GXXNF53-1805	Guangxi	2018	L3
ON462046	GXXNF10-1803	Guangxi	2018	L3
ON462045	GXTZJ2325-2112	Guangxi	2021	L3
ON462044	HNTZJ1714-2011	Henan	2020	L3
ON462043	HNLCL15-1903	Henan	2019	L1
ON365556.1	CN/SS0/2020	Fujian	2020	L8
ON254651.1	SD	Heilongjiang	2016	L1
ON254650.1	SD-R	Heilongjiang	2017	L1
ON180781	YC-2020	Shanxi	2021	L1
ON093974.1	CN/SS1/2021	Fujian	2021	L8
OM949993.1	GDqy-1909	Guangdong	2019	L8
OM949992.1	GDhy-1809	Guangdong	2018	L1
OM293962.1	HN0713	Henan	2021	L1
OM293961.1	GDYJ1224	Guangdong	2020	L1
OM293960.1	GDHY0425	Guangdong	2021	L8
OM293959.1	GDGZ0408	Guangdong	2021	L8
OM202903.1	GX11373	Guangxi	2020	L8
OM202902.1	GX11045	Guangxi	2020	L8
OM202901.1	GX7668	Guangxi	2020	L8
OM202900.1	GX7111	Guangxi	2020	L8
OM202899.1	GX5416	Guangxi	2021	L8
OM202898.1	GX4934	Guangxi	2021	L8
OM202897.1	GX4852	Guangxi	2021	L1
OM202896.1	GX3251	Guangxi	2021	L8
OM202895.1	GX1858	Guangxi	2020	L1
OM202894.1	GX505	Guangxi	2020	L1
OM202893.1	GD7666	Guangdong	2020	L8
OM201199.1	S032	Shandong	2018	L8
OM201198.1	S022	Shandong	2018	L8
OM201197.1	S020	Shandong	2018	L1
OM201196.1	S001	Shandong	2018	L1
OM201195.1	LN86	Liaoning	2018	L1
OM201194.1	HB96	Hebei	2019	L5
OM201193.1	HB94	Hebei	2019	L1
OM201192.1	H64	Heilongjiang	2019	L1
OM201191.1	H60	Hebei	2018	L1
OM201190.1	PRRSV/H029	Heilongjiang	2018	L1
OM201189	H013	Hebei	2018	L1
OM201188.1	H012	Hebei	2018	L1
OM201187.1	G128	Guangdong	2018	L3
OM201186.1	G122	Guangdong	2018	L8
OM201185.1	G101	Guangdong	2019	L8
OM201184.1	G59	Guangdong	2018	L3
OM201183.1	G52	Guangdong	2018	L8
OM201182.1	G113	Guangdong	2018	L3
OM201181.1	ZJ83	Zhejiang	2018	L3

OM201180	S145	Shandong	2019	L1
OM201179.1	S136	Shandong	2019	L1
OM201178.1	S130	Shandong	2019	L1
OM201177.1	S78	Shandong	2018	L1
OM201176.1	S77	Shandong	2018	L1
OM201175.1	S75	Shandong	2018	L8
OM201174.1	S70	Shandong	2018	L3
OM201173.1	S043	Shandong	2018	L1
OM201172.1	S039	Shandong	2018	L1
OM201171.1	C103	Chongqing	2019	L5
OM141114	CN/L5	Fujian	2018	L1
OL771209	CH/SCYB-2/2020	Sichuan	2020	L1
OL771208	CH/SCMS-4/2020	Sichuan	2020	L1
OL771207	CH/SCLS-2/2020	Sichuan	2020	L1
OL771206	CH/SCCD-4/2020	Sichuan	2020	L1
OL771205	CH/SCMY-2/2019	Sichuan	2019	L1
OL687155	HY21	Hunan	2021	L8
OL516361	SDHSW135-2009	Shandong	2020	L1
OL516360	LNTZJ1341-2012	Liaoning	2020	L1
OL516359	JLTZJ2050-2107	Jilin	2021	L1
OL516358	HNTZJ165-2001	Henan	2020	L1
OL516357	HLJWK318-2001	Heilongjiang	2020	L1
OL516356	HLJTZJ2165-2108	Heilongjiang	2021	L1
OL516355	HLJTZJ2090-2107	Heilongjiang	2021	L1
OL516354	HLJTZJ2007-2106	Heilongjiang	2021	L1
OL516353	HLJTZJ1988-2106	Heilongjiang	2021	L1
OL516352	HLJTZJ1289-2012	Heilongjiang	2020	L1
OL516351	HLJTZJ921-2011	Heilongjiang	2020	L1
OL516350	HLJTZJ864-2010	Heilongjiang	2020	L1
OL516349	HLJTZJ829-2010	Heilongjiang	2020	L1
OL516348	HLJPY32-2109	Heilongjiang	2021	L1
OL516347	HLJPY18-2009	Heilongjiang	2020	L1
OL439476	GXGG202007	Guangxi	2020	L1
OL422844	CN/Z0	Fujian	2021	L1
OL422843	CN/Q9	Fujian	2021	L1
OL422842	CN/N0	Fujian	2021	L1
OL422841	CN/N2	Fujian	2021	L1
OL422840	CN/J2	Fujian	2019	L1
OL422839	CN/H5	Fujian	2020	L8
OL422838	CN/H2	Fujian	2020	L8
OL422837	CN/F3	Fujian	2020	L8
OL422836	CN/F2	Fujian	2019	L1
OL422835	CN/L2	Fujian	2019	L8
OL422834	CN/L1	Fujian	2019	L8
OL422833	CN/C2	Fujian	2019	L8
OL422832	CN/F0	Fujian	2018	L1
OL422831	CN/Z8	Fujian	2018	L1
OL422830	CN/F7	Fujian	2017	L1
OL422829	CN/S5/2018	Fujian	2018	L1
OL422828	CN/N42/2017	Fujian	2017	L1
OL422827	CN/N3/2017	Fujian	2017	L1
OL422826	CN/J8/2018	Fujian	2018	L8
OL422825	CN/H4/2018	Fujian	2018	L8
OL422824	CN/F8/2020	Fujian	2020	L8
OL422823	CN/N4/2019	Fujian	2019	L1
OL422822	CN/L4/2020	Fujian	2020	L8
OL416130	CN/L3/2021	Fujian	2021	L8
OL416129	CN/I9/2018	Fujian	2019	L1
OL416128	CN/H1/2018	Fujian	2019	L1
OL416127	CN/G9/2018	Fujian	2019	L1
OL416126.1	CN/G7/2018	Fujian	2018	L1
OL416125	CN/F5/2018	Fujian	2018	L1
OL416124	CN/E9/2018	Fujian	2018	L1
OL310959	CN/FJGD01/2021	Fujian	2021	L1
OK486524	GXNN20210506	Guangxi	2021	L1
OK486523	GXQZ20210403	Guangxi	2021	L1
OK486522	GXFCG20210401	Guangxi	2021	L1
OK274266	ZJqz21	Zhejiang	2021	L1
OK095299	BJ2021	Beijing	2021	L3
MZ820388	JS2021NADC34	Jiangsu	2021	L1
MZ747449.1	CH/SCZG	Sichuan	2016	L8
MZ747448.1	CH/SCYB	Sichuan	2019	L1
MZ747447.1	CH/SCYB-1	Sichuan	2018	L1
MZ747446.1	CH/SCYA-1	Sichuan	2019	L1
MZ747445	CH/SCSN	Sichuan	2017	L8

MZ747444.1	CH/SCPZ	Sichuan	2020	L1
MZ747443.1	CH/SCNC-2	Sichuan	2020	L1
MZ747442.1	CH/SCMY-3	Sichuan	2020	L1
MZ747441.1	CH/SCMS-3	Sichuan	2019	L8
MZ747440.1	CH/SCMS-1	Sichuan	2016	L8
MZ747439.1	CH/SCHY	Sichuan	2018	L1
MZ747438.1	CH/SCGY-2	Sichuan	2020	L1
MZ747437.1	CH/SCCD-2	Sichuan	2018	L1
MZ712110.1	HB2104	Hebei	2021	L1
MZ579701	HBap4/2018	Hubei	2018	L8
MZ540774	PRRSVM	Zhejiang	2017	L8
MZ399801.1	TA-12	Jiangsu	2007	L8
MZ399800	NL1207	Jiangsu	2019	L1
MZ342900	JS2020	Jiangsu	2020	L1
MZ322956	SDRZ01	Shandong	2019	L8
MZ219272	CH/GX	Guangxi	2019	L8
MZ219271.1	CH/GX/2475	Guangxi	2019	L8
MZ219270.1	CH/GX/2283	Guangxi	2018	L8
MZ219269	CH/GX/PRRSV/2271/2018	Guangxi	2018	L8
MZ172971	SD-QD-2101	Shandong	2020	L8
MZ169406	YL-2021	Shanxi	2021	L8
MZ160905.1	PRRSV NA	Shaanxi	2020	L1
MZ146722.1	FJ1805	Fujian	2018	L1
MZ146721.1	SD1805	Shandong	2018	L1
MZ047781.1	SDlz20-04	Hebei	2020	L1
MZ047780.1	BJ20-06	Hebei	2020	L1
MZ043753.1	CHN-HB	Hubei	2018	L1
MW880772	SXSZ-2020	Shanxi	2020	L1
MW853923	AH-PRRS20178-1	Anhui	2017	L8
MW822587.1	ZJ-JX-2015	Zhejiang	2015	L8
MW803134	SCcd2020	Sichuan	2020	L1
MW651976	HB19-18	Hubei	2019	L8
MW651975	HB19-12	Hubei	2019	L8
MW627193	HB18-36	Hubei	2018	L8
MW561594	GXNN202004	Guangxi	2020	L1
MW561593	GXNN202010	Guangxi	2020	L3
MW531679	GXNN202004a	Guangxi	2020	L1
MW408254	SD 2020	Guizhou	2020	L8
MW079495	2020-Acheng-1	Jilin	2020	L1
MT811841	YNML2018	Yunnan	2018	L8
MT811840	YNXW2017	Yunnan	2017	L8
MT811839	YNXS2017	Yunnan	2017	L8
MT811838	YNPL2016	Yunnan	2016	L8
MT811837	YNSB2016	Yunnan	2017	L8
MT811836	YNSM2016	Yunnan	2016	L8
MT811835	YNSL2018	Yunnan	2018	L8
MT811834	YNSD2017	Yunnan	2017	L8
MT811833	YNQJ2017	Yunnan	2017	L8
MT811832	YNQJXW2017	Yunnan	2017	L8
MT811831	YNLQ2016	Yunnan	2016	L8
MT811830	YNYL2016	Yunnan	2017	L8
MT811829	YNJN2016	Yunnan	2016	L8
MT811828	YNDL2016	Yunnan	2017	L8
MT811827	YNCX2016	Yunnan	2016	L8
MT811826	YNCXZX2017	Yunnan	2017	L8
MT811825	YNCL2018	Yunnan	2018	L8
MT811824	YNWH2016	Yunnan	2017	L8
MT811823	YNCN2017	Yunnan	2017	L8
MT811822	YNAN2018	Yunnan	2018	L8
MT780871	JSYZ1909-16	Jiangsu	2019	L8
MT746146	JSYC20-05-1	Jiangsu	2020	L5
MT721741	PRRSV2/CN/GDDX/2018	Guangdong	2018	L8
MT708500	SD-YL1712	Shandong	2017	L8
MT663768	TS01	na	2018	L8
MT416548	PRRSV2/CN/101805/2018	Fujian	2018	L5
MT416547.1	FJDJQ-2018	Fujian	2018	L1
MT416546	PRRSV2/CN/110713/2018	Fujian	2018	L1
MT416545	PRRSV2/CN/101806/2018	Fujian	2018	L8
MT416544	PRRSV2/CN/F1004/2017	Fujian	2017	L3
MT416543	PRRSV2/CN/F1228/2017	Fujian	2017	L8
MT416542	PRRSV2/CN/N9185/2018	Fujian	2018	L3
MT416541	PRRSV2/CN/X2984/2018	Fujian	2018	L8
MT409692	PRRSV2/CN/X4839/2017	Fujian	2017	L1
MT409691	PRRSV2/CN/X4833/2018	Fujian	2018	L1
MT409690	PRRSV2/CN/X2998/2018	Fujian	2018	L8

MT409689	PRRSV2/CN/X4836/2018	Fujian	2018	L1
MT409688	PRRSV2/CN/X9830/2018	Fujian	2018	L1
MT409687	PRRSV2/CN/X4831/2018	Fujian	2018	L1
MT394497	GDsc1809	Guangdong	2018	L1
MT394496	GDsc1808	Guangdong	2018	L1
MT394495	GDxn1808	Guangdong	2018	L1
MT394494	GDhh1808	Guangdong	2018	L8
MT379661	GDDX-2018	Guangdong	2018	L8
MT316312	HB18-4	Hubei	2019	L8
MT268280	HB18-41	Hubei	2018	L8
MT165636	GD1909	Guangdong	2019	L1
MT093771	SD217-1705	Shandong	2017	L5
MT093770	SD192-1703	Shandong	2017	L5
MT093769	SD160-1612	Shandong	2016	L5
MT093768	SD158-1612	Shandong	2016	L5
MT093767	SD218-1706	Shandong	2017	L3
MT093766	SD136-1609	Shandong	2016	L3
MT093765	SD130-1609	Shandong	2016	L3
MT093764	SD265-1801	Shandong	2018	L1
MT093763	SD288-1805	Shandong	2018	L1
MT093762	SD261-1801	Shandong	2018	L1
MT093761	SD167-1702	Shandong	2017	L1
MT093760	SD157-1612	Shandong	2016	L1
MT093759	SD85-1605	Shandong	2016	L1
MT093758	SD303-1806	Shandong	2018	L1
MT093757	SD254-1712	Shandong	2017	L1
MT093756	SD235-1711	Shandong	2017	L1
MT093755	SD281-1805	Shandong	2018	L1
MT093754	SD245-1712	Shandong	2017	L1
MT093753	SD205-1704	Shandong	2017	L1
MT093752	SD191-1703	Shandong	2017	L1
MT093751	SD176-1702	Shandong	2017	L1
MT093750	SD171-1702	Shandong	2017	L1
MT093749	SD91-1605	Shandong	2016	L1
MT093748	SD79-1605	Shandong	2016	L1
MT093747	SD75-1605	Shandong	2016	L1
MT093746	SD70-1605	Shandong	2016	L1
MT093745	SD56-1603	Shandong	2016	L1
MT093744	SD180-1702	Shandong	2017	L8
MT093743	SD173-1702	Shandong	2017	L8
MT093742	SD172-1702	Shandong	2017	L8
MT093741	SD65-1603	Shandong	2016	L8
MT093740	SD54-1603	Shandong	2016	L8
MT093739	SD23-1505	Shandong	2015	L8
MT075480	SC/DJY	Sichuan	2019	L1
MT036900	XY-HN_Xinyang	Henan	2017	L1
MT036899	HZ1-3_Yunnan_20171	Yunnan	2017	L1
MT036898	GDHZ_Huizhou	Guangdong	2017	L1
MT036897	FS-GD-02	Guangdong	2016	L1
MN862433	FJDJQ-2018	Fujian	2018	L1
MN660070	GXNN1839	Guangxi	2018	L1
MN660069	GXYL1403	Guangxi	2014	L8
MN660067	GXNN1396-p3	Guangxi	2013	L8
MN648450	HLJZD22-1812	Heilongjiang	2018	L1
MN648449	HLHDZD32-1901	Heilongjiang	2019	L1
MN648055	HLJZD30-1902	Heilongjiang	2019	L1
MN648054	LNDZD10-1806	Liaoning	2018	L1
MN642105	SDyt1401	Shandong	2014	L1
MN642104	SDwh1701	Shandong	2017	L5
MN642103	SDwh1602	Shandong	2016	L8
MN642102	SDwh1601	Shandong	2016	L1
MN642101	SDwh1403	Shandong	2014	L1
MN642100	SDwh1402	Shandong	2014	L8
MN642099	SDqd1501	Shandong	2015	L3
MN606305	JX07	Jiangxi	2007	L8
MN606304	JS18-3	Jiangsu	2018	L1
MN547967	JSTZ1907-714	Jiangsu	2019	L8
MN547966	JSTZ1904-664	Jiangsu	2019	L8
MN547965	JSTZ1810-220	Jiangsu	2018	L1
MN547964	JS1703-21	Jiangsu	2017	L8
MN401750	SW2018001-YL	Taiwan	2018	L3
MN119309	XJ1904-39	Xinjiang	2019	L1
MN119308	JS1810-195	Jiangsu	2018	L8
MN119307	HN1804-2	Henan	2018	L1
MN119306	SH1704-25	Shanghai	2017	L8

MN119305	SD1704-23	Shandong	2017	L1
MN119304	SD1612-1	Shandong	2016	L8
MN046243	HLJ-DZD4-1805	Heilongjiang	2018	L1
MN046242	LN-DB87	Liaoning	2018	L3
MN046241	SX2-1607	Shanxi	2016	L1
MN046240	SX1-1607	Shanxi	2016	L1
MN046239	HLJ-YC8	Heilongjiang	2018	L8
MN046238	HN-1603	Hunan	2016	L8
MN046237	InterMo-2015-2	Inner Mongolia	2015	L8
MN046236	Sichuan-2017-117	Sichuan	2017	L8
MN046235	Gansu-2017-51	Gansu	2017	L8
MN046234	SDJM-1602	Shandong	2016	L8
MN046233	Liaoning-2017-6	Liaoning	2017	L8
MN046232	JS3-1805	Jiangsu	2018	L8
MN046231	Anhui-2017-109	Anhui	2017	L8
MN046230	HLJWK108-1711	Heilongjiang	2018	L1
MN046229	HeB-239	Hebei	2018	L1
MN046228	HeNXX-2014-12	Henan	2014	L1
MN046227	HeNXX-2014-9	Henan	2014	L8
MN046226	HeNXX-2014-3	Henan	2014	L1
MN046225	Fujian-2014-18	Fujian	2014	L1
MN046224	HEB-108	Hebei	2017	L1
MN046223	HLJ-DZD1-1804	Heilongjiang	2018	L8
MN046222	HLJ-80	Heilongjiang	2016	L1
MN046221	2014-81	Heilongjiang	2014	L8
MN026347	NM-12	Jilin	2019	L8
MN026346	GXBB16-1	Guangxi	2016	L8
MK906026	JSTZ1712-12	Jiangsu	2017	L8
MK780825	SD110-1608	Shandong	2016	L3
MK780824	SDWH27-1710	Shandong	2017	L3
MK759853	XJ17-5	Xinjiang	2017	L8
MK453050	ZDXYL-China-2018-2	Heilongjiang	2018	L1
MK453049	ZDXYL-China-2018-1	Heilongjiang	2018	L1
MK450365	CH-YY	na	2018	L8
MK450333	CH-WH-2019-1	Hubei	2018	L1
MK429987	SWU/CQ1/2018	Chongqing	2018	L5
MK429986	SWU/CD1/2018	Sichuan	2018	L1
MK429985	SWU/YB2/2018	Sichuan	2018	L1
MK429984	SWU/YB1/2018	Sichuan	2018	L1
MK429983	SWU/MY6/2018	Sichuan	2018	L1
MK429982	SWU/MY5/2018	Sichuan	2018	L1
MK429981	SWU/MS3/2018	Sichuan	2018	L1
MK429980	SWU/MS2/2018	Sichuan	2018	L1
MK396384	GDsf1809	Guangdong	2018	L1
MK396383	GDsf1808	Guangdong	2018	L1
MK396382	GDsf1807	Guangdong	2018	L1
MK396381	GDsf1806	Guangdong	2018	L1
MK396380	GDsf1804	Guangdong	2018	L1
MK396379	GDsf1802	Guangdong	2018	L1
MK396378	GDsf1711	Guangdong	2017	L1
MK396377	GDsf1710	Guangdong	2017	L1
MK396376	GDsf1707	Guangdong	2017	L1
MK279741	CHN/JK/201805	Hubei	2018	L1
MK279740	CHN/TG/201711	Hubei	2017	L8
MK279739	CHN/JTS/201606	Hubei	2016	L8
MK202794	FJ0908	Fujian	2018	L1
MK144543	SCya18	Sichuan	2018	L3
MK144542	GZgy17	Guizhou	2017	L3
MH663433	HNRZ	Hunan	2017	L8
MH651748	TJZH-1607	Tianjin	2016	L1
MH651747	SDZC-1609	Shandong	2016	L8
MH651746	SDQZ-1609	Shandong	2016	L1
MH651745	SD99-1606	Shandong	2016	L1
MH651744	SD53-1603	Shandong	2016	L1
MH651743	SD-1602	Shandong	2016	L1
MH651742	SDQD-1604	Shandong	2016	L1
MH651741	LNCH-1604	Liaoning	2016	L1
MH651740	HNJYH-1606	Hunan	2016	L1
MH651739	HBFL-1604	Hubei	2016	L1
MH651738	HNJYF-1606	Hunan	2016	L1
MH651737	CY2-1604	Heilongjiang	2016	L1
MH651736	CY1-1604	Heilongjiang	2016	L1
MH588710	SDbz16-2	Shandong	2016	L1
MH500776.1	NADC30	Shandong	2017	L1
MH404256	SD17	Shandong	2017	L8

MH370474	CH/2018/NCV-Anheal-1	na	2018	L1
MH324400	SCya17	Sichuan	2017	L3
MH236426	ZJnb16-2	Zhejiang	2016	L3
MH167388	QHD3	Qinghai	2017	L1
MH167387	QHD2	Qinghai	2017	L1
MH121061	SD17-36	Guangdong	2017	L1
MH078490	SCN17	Sichuan	2017	L1
MH068878	SD17-38	Shandong	2017	L1
MH046843	GDZS2016	Guangdong	2016	L3
MH046842	FJNP2017	Fujian	2017	L3
MG914067	SCcd17	Sichuan	2017	L1
MG913987	LNWK130	Liaoning	2017	L1
MG860516	LNWK96	Liaoning	2017	L1
MG844181	HB17A	Hebei	2017	L1
MG687491	QHD1	Qinghai	2017	L1
MG011719	FJDJQ-2017	Fujian	2017	L3
MG011718	FJLIUY-2017	Fujian	2017	L3
MF818049	SC/NJ 2016	Sichuan	2016	L8
MF772778	GDzj	Guangdong	2017	L8
MF770574	17-ZJ-HZ	Zhejiang	2017	L8
MF766474	HeN1601	Henan	2016	L1
MF766473	HeN1502	Henan	2015	L8
MF766472	HeN1501	Henan	2015	L8
MF766471	HeN1401	Henan	2014	L1
MF766470	HeN1301	Henan	2013	L8
MF689000	HeN1201	Henan	2012	L8
MF669722	ZJXS1412	Zhejiang	2014	L8
MF669721	HZL1501	na	2015	L8
MF669720	GD1404	Guangdong	2014	L8
MF526896	GDQYQC2	Guangdong	2016	L8
MF375261	SC-d	Sichuan	2015	L1
MF375260	SD-A19	Shandong	2015	L1
MF370557	FZ06A	Fujian	2006	L8
MF196906	SCnj16	Sichuan	2016	L1
MF196905	SCcd16	Sichuan	2016	L3
MF124329	GD1404	Guangdong	2014	L3
KY761966	FZ16A	Fujian	2016	L8
KY745901	GDYDZZZ	Guangdong	2016	L3
KY498542	GDST	Guangdong	2014	L8
KY495781	SH/CH/2016	Shanghai	2016	L3
KY495780	JX/CH/2016	Jiangxi	2016	L3
KY488479	GDSG	Guangdong	2017	L8
KY488478	GDQY	Guangdong	2015	L8
KY488477	GDHZ	Guangdong	2015	L8
KY488476	GDHY	Guangdong	2015	L8
KY488475	HNHK1	Hunan	2014	L8
KY488474	HNHK2	Hunan	2014	L8
KY488473	GDZQ	Guangdong	2014	L8
KY488472	GDMM	Guangdong	2014	L8
KY488471	GDGZ	Guangdong	2014	L8
KY488470	GDJM	Guangdong	2014	L8
KY412888	FJM4	Fujian	2014	L1
KY412887	FJL15	Fujian	2014	L1
KY373218	SXF105	Shanxi	2014	L8
KY373217	SDZZ	Shandong	2014	L8
KY373216	AHBZ	Anhui	2014	L8
KY373215	HiNZWQ	Hainan	2014	L3
KY373214	JSWA	Jiangsu	2014	L1
KY290748	HENXX-9	Henan	2016	L8
KY053458	SDYG1606	Shandong	2016	L1
KY041782	HENXX-8	Henan	2016	L1
KX980393	SDlz1601	Shandong	2016	L8
KX980392	SDhz1512	Shandong	2015	L1
KX900392	HENJY-2	Henan	2015	L1
KX815434	15ZJ3	Zhejiang	2015	L8
KX815433	15ZJ2	Zhejiang	2015	L8
KX815432	15ZJ1	Zhejiang	2015	L1
KX815431	15SN3	Shannxi	2015	L8
KX815430	15SN2	Shannxi	2015	L8
KX815429	15SN1	Shannxi	2015	L8
KX815428	15SC3	Sichuan	2015	L1
KX815427	15SC2	Sichuan	2015	L8
KX815426	15SC1	Sichuan	2015	L8
KX815425	15LN3	Liaoning	2015	L1
KX815424	15LN2	Liaoning	2015	L8

KX815423	15LN1	Liaoning	2015	L1
KX815422	15JX4	Jiangxi	2015	L8
KX815421	15JX3	Jiangxi	2015	L8
KX815420	15JX2	Jiangxi	2015	L8
KX815419	15JX1	Jiangxi	2015	L1
KX815418	15HUN3	Hunan	2015	L8
KX815417	15HUN2	Hunan	2015	L8
KX815416	15HUN1	Hunan	2015	L8
KX815415	15HEN4	Henan	2015	L1
KX815414	15HEN3	Henan	2015	L8
KX815413	15HEN1	Henan	2015	L1
KX815412	15HEB3	Hebei	2015	L8
KX815411	15HEB1	Hebei	2015	L1
KX815410	15GD4	Guangdong	2015	L8
KX815409	15GD3	Guangdong	2015	L8
KX815408	15GD2	Guangdong	2015	L8
KX815407	15GD1	Guangdong	2015	L8
KX767091	GSWW/CHA 2015	Gansu	2015	L8
KX766379	HNhx	Hunan	2016	L1
KX766378	HN07-1	Hunan	2007	L8
KX758250	FJXS15	Fujian	2015	L1
KX758249	FJWQ16	Fujian	2016	L1
KX689233	XJzx1-2015	Xinjiang	2015	L3
KX650082	RVB-581	na	2008	L8
KX621003	GDsg	Guangdong	2015	L3
KX510269	TJnh1501	Tianjin	2015	L8
KX357708	QTX	na	2016	L8
KX169191	FJ1402	Fujian	2014	L1
KU978619	GD-KP	Guangdong	2015	L3
KU950375	HENZZ-8	Henan	2015	L8
KU950374	HENZMD-9	Henan	2015	L1
KU950373	HENZK-1	Henan	2014	L8
KU950372	HENXX-1	Henan	2014	L1
KU950371	HENXC-4	Henan	2015	L1
KU950370	HENPDS-2	Henan	2015	L8
KU523367	WUH6	Hubei	2011	L1
KU523366	WUH5	Hubei	2015	L1
KU215417	15LY02-FJ	Fujian	2015	L8
KU215416	15LY01-FJ	Fujian	2015	L8
KU201579	QH-08	Qinghai	2008	L8
KT945018	HNyc15	Henan	2015	L3
KT945017	HNjz15	Henan	2015	L1
KT819203	SCwhn14DY	Sichuan	2014	L8
KT804696	FJYR	Fujian	2015	L8
KT445876	HNP5	na	2014	L8
KT358728	GZgy15-1	Guizhou	2015	L8
KT351740	HLJB1	Heilongjiang	2013	L8
KT351739	HLJA1	Heilongjiang	2013	L8
KT180169	XF1129	na	2013	L8
KT033733.1	QH-1(sh)/2008	Gansu	2008	L8
KT022072	HNyc13	Hunan	2013	L8
KT022071	HNxa14	Hunan	2014	L8
KR706343	JL580	Jilin	2013	L1
KR149645	JXja15	Jiangxi	2015	L8
KP998479	FJOU	Fujian	2015	L8
KP998478	FJZH	Fujian	2015	L8
KP998477	FJCH	Fujian	2015	L8
KP998476	FJFS	Fujian	2015	L3
KP998475	FJE1	Fujian	2015	L8
KP998474	FJSD	Fujian	2015	L5
KP998431	MD001	Taiwan	1991	L3
KP998430	HC120629	Taiwan	2012	L3
KP998429	WSV	Taiwan	1992	L5
KP998427	TD-2	Taiwan	2004	L3
KP998426	CH8V-J2	Taiwan	2003	L3
KP998425	TD1	Taiwan	1997	L3
KP998424	TY1	Taiwan	2000	L3
KP998423	Tsai	Taiwan	1999	L3
KP998422	TD/TP	Taiwan	1998	L3
KP998421	Q94-136	Taiwan	2005	L3
KP998420	NT	Taiwan	2000	L3
KP998419	M1	Taiwan	2001	L3
KP998418	HL	Taiwan	2004	L3
KP998416	CH	Taiwan	2002	L3
KP998415	803	Taiwan	2013	L3

KP998414	660	Taiwan	2009	L3
KP998413	310	Taiwan	2005	L3
KP998412	25934	Taiwan	2008	L3
KP998410	JM	Taiwan	2011	L3
KP998409	HC120904-CHYL	Taiwan	2012	L3
KP998408	HC120821-LL	Taiwan	2012	L3
KP998407	HC120821-SH2	Taiwan	2012	L3
KP998406.1	HC120821-SH1	Taiwan	2012	L3
KP998404	338	Taiwan	2011	L3
KP998403	1483	Taiwan	2012	L3
KP998402	312	Taiwan	2005	L3
KP998401	17199	Taiwan	2005	L3
KP861625	CHsx1401	Shanxi	2014	L1
KP860911	FJW05	Fujian	2015	L1
KP860910	FJY04	Fujian	2015	L1
KP860909	FJZ03	Fujian	2015	L1
KP793736	GD-HD	Guangdong	2011	L8
KP780882	14LY02-FJ	Fujian	2014	L8
KP780881	14LY01-FJ	Fujian	2014	L8
KP771784	NVDC-SD4-2014	Shandong	2014	L8
KP771783	NVDC-R224-2014	Beijing	2014	L8
KP771782	NVDC-R38-2014	Beijing	2014	L8
KP771781	NVDC-HuNCS-2014	Hunan	2014	L8
KP771780	NVDC-13SXJC-2014	Shanxi	2014	L8
KP771779	NVDC-NM-2008	Inner Mongolia	2008	L8
KP771778	NVDC-BJ1-2011	Beijing	2011	L8
KP771777	NVDC-CQ4-2012	Chognqing	2012	L8
KP771776	NVDC-CQ2-2012	Chongqing	2012	L8
KP771775	NVDC-CQ3-2012	Chongqing	2012	L8
KP771774	NVDC-CQ3-2011	Chongqing	2011	L8
KP771773	NVDC-HeB1-2012	Hebei	2012	L8
KP771772	NVDC-HeB2-2012	Hebei	2012	L8
KP771771	NVDC-HeN-2012	Henan	2012	L8
KP771770	NVDC-HuN-2011	Hunan	2012	L8
KP771769	NVDC-SD1-2012	Shandong	2012	L8
KP771768	NVDC-SD2-2012	Shandong	2012	L8
KP771767	NVDC-YN-2011	Yunnan	2011	L8
KP771766	NVDC-GD-2011	Guangdong	2011	L8
KP771765	NVDC-HeB2-2011	Hebei	2011	L8
KP771764	NVDC-BJ1-2012	Beijing	2012	L8
KP771763	NVDC-BJ2-2012	Beijing	2012	L8
KP771762	NVDC-BJ3-2012	Beijing	2012	L8
KP771761	NVDC-BJ4-2012	Beijing	2012	L8
KP771760	NVDC-BJ5-2012	Beijing	2012	L8
KP771759	NVDC-BJ6-2012	Beijing	2012	L8
KP771758	NVDC-BJ7-2012	Beijing	2012	L8
KP771757	NVDC-BJ8-2012	Beijing	2012	L8
KP771756	NVDC-BJ9-2012	Beijing	2012	L8
KP771755	NVDC-CQ-2008	Chongqing	2008	L8
KP771754	NVDC-HeB-2008	Hebei	2008	L8
KP771753	HEB 20130008-13	Hebei	2013	L8
KP771752.1	HEB 20130008-14	Hebei	2013	L8
KP771751	NVDC-MD1-2013	Beijing	2013	L8
KP771750	NVDC-MD2-2013	Beijing	2013	L8
KP771749	NVDC-HeB1-2011	Hebei	2011	L8
KP771748	NVDC-BJ2-2011	Beijing	2011	L8
KP771747	NVDC-CQ1-2012	Chongqing	2012	L8
KP771746	NVDC-CQ1-2011	Chongqing	2011	L8
KP771745	NVDC-HeB1-2013	Hebei	2013	L8
KP771744	NVDC-HeB2-2013	Hebei	2013	L8
KP771743	NVDC-BJPG-2013	Beijing	2013	L8
KP771742	NVDC-HBCZ-2013	Hubei	2013	L8
KP771741	NVDC-SDXX-2013	Shandong	2013	L8
KP771740	NVDC-SXJC-2013	Shanxi	2013	L8
KP771739	NVDC-SC1-2014	Sichuan	2014	L8
KP771738	NVDC-SD1-2014	Shandong	2014	L8
KP771737	NVDC-SD6-2014	Shandong	2014	L8
KP771736	NVDC-shh01-2014	Shanghai	2014	L8
KP771735	NVDC-SHH02-2014	Shanghai	2014	L8
KP742987	TJbd14-2	Tianjin	2014	L8
KP742986	TJbd14-1	Tianjin	2014	L8
KP330232	HUN-2014	Hunan	2014	L8
KP179404	NT3	Jiangsu	2012	L8
KP179403	NT2	Jiangsu	2012	L8
KP179402	NT1	Jiangsu	2012	L8

KP162169	HB-XL	na	2014	L8
KM261784	HB2014001	Hubei	2014	L8
KM189443	SC2012	Sichuan	2012	L8
KM000066	NMG2014	Inner Mongolia	2014	L8
KJ855518	Shanxi-6	Shanxi	2010	L8
KJ819936	Henan-A14	Henan	2014	L8
KJ819935	Henan-A13	Henan	2014	L8
KJ819934	Henan-A12	Henan	2014	L8
KJ747052	YN-1	Yunnan	2011	L8
KJ609517	MY-376	Henan	2013	L8
KJ609516	MY-486	Henan	2013	L8
KJ591659	HEB-2013	Hebei	2013	L8
KJ546412	HeNan-A9	Henan	2013	L8
KJ541663	GZ106	Guizhou	2005	L8
KJ534543	Henan-A8	Henan	2013	L8
KJ534542	Henan-A7	Henan	2013	L8
KJ534541	Henan-A6	Henan	2013	L8
KJ534540	Henan-A5	Henan	2013	L8
KJ534539	Henan-A4	Henan	2013	L8
KJ143621	HENAN-HEB	Henan	2012	L1
KJ019330	Henan-A3	Henan	2013	L8
KJ002452	HeNan-A2	Henan	2013	L8
KJ002451	HeNan-A1	Henan	2013	L8
KF815525	XJu-1	Xinjiang	2012	L8
KF771273	GZ1101	Guizhou	2011	L5
KF751238	LN1101	Liaoning	2011	L8
KF751237	BJ1102	Beijing	2011	L8
KF678434	SH1211	Shanghai	2012	L3
KF611905	HENAN-XINX	Henan	2013	L1
KF287143	HK16	Hongkong	2004	L3
KF287142	HK15	Hongkong	2004	L3
KF287141	HK14	Hongkong	2004	L5
KF287140	HK13	Hongkong	2005	L8
KF287139	HK12	Hongkong	2004	L8
KF287138	HK11	Hongkong	2004	L3
KF287137	HK9	Hongkong	2004	L3
KF287136	HK7	Hongkong	2004	L8
KF287135	HK6	Hongkong	2004	L3
KF287134	HK4	Hongkong	2003	L8
KF287133	HK2	Hongkong	2003	L3
KF287132	HK1	Hongkong	2003	L8
KC527830	GD-2011	Guangdong	2011	L8
KC445138	HZ-31	na	2012	L5
JX912249	GX1003	Guangxi	2010	L8
JX880029	NJ-1106	Jiangsu	2011	L8
JX878380	SDA3	Shandong	2011	L8
JX878379	SDA2	Shandong	2011	L8
JX857698	YN-2011	Yunnan	2011	L5
JX679179	HH08	Heilongjiang	2011	L8
JX317649	JX	Jiangxi	2010	L8
JX317648	HV	na	2007	L8
JX235370	11GZ-GD	Guangdong	2011	L8
JX235367	11XX-GD	Guangdong	2011	L8
JX235366	11SH1-GD	Guangdong	2011	L8
JX235365	11SH-GD	Guangdong	2011	L8
JX217036	11NZ-GD	Guangdong	2011	L8
JX215554	11FS12-GD	Guangdong	2011	L8
JX215553	10HD-GD	Guangdong	2010	L8
JX215552	10QY-GD	Guangdong	2010	L8
JX215551	11FS11-GD	Guangdong	2011	L8
JX192639	10ZQ-GD	Guangdong	2010	L8
JX192638	10SS-GD	Guangdong	2010	L8
JX192637	10SJ-GD	Guangdong	2010	L8
JX192636	10BY-GD	Guangdong	2010	L8
JX192635	10FS1-GD	Guangdong	2010	L8
JX192634	10FS-GD	Guangdong	2010	L8
JX192633	10GZ-GD	Guangdong	2010	L8
JX192632	10HN-GD	Guangdong	2010	L8
JX177644	JL-04/12	Jilin	2012	L8
JX087437	SD16	Shandong	2012	L8
JQ955658	GX1002	Guangxi	2010	L8
JQ955657	GX1001	Guangxi	2010	L8
JQ743666	QY2010	Guangdong	2010	L3
JQ715698	NVDC-JS2-2011	Jiangsu	2011	L8
JQ715697	NVDC-GD2-2011	Guangdong	2011	L8

JQ663562	10-10GX-5	Guangxi	2010	L8
JQ663561	10-10GX-4	Guangxi	2010	L8
JQ663560	10-10GX-3	Guangxi	2010	L8
JQ663559	10-10GX-2	Guangxi	2010	L8
JQ663558	10-10GX-1	Guangxi	2010	L8
JQ663556	10-10QN	na	2010	L8
JQ663555	10-10SD	Shandong	2010	L8
JQ663554	10-10JL	Jilin	2010	L8
JQ663553	10-10HEB-3	Hebei	2010	L8
JQ663552	10-10HEB-2	Hebei	2010	L8
JQ663551	10-10HEB-1	Hebei	2010	L8
JQ663550	10-10FUJ-5	Fujian	2010	L8
JQ663549	10-10FUJ-4	Fujian	2010	L8
JQ663548	10-10FUJ-3	Fujian	2010	L8
JQ663547	10-10FUJ-2	Fujian	2010	L8
JQ663546	10-10FUJ-1	Fujian	2010	L8
JQ663545	10-10BJ-5	Beijing	2010	L8
JQ663544	10-10BJ-4	Beijing	2010	L8
JQ663543	10-10BJ-2	Beijing	2010	L8
JQ663542	10-10BJ-3	Beijing	2010	L8
JQ663541	10-10BJ-1	Beijing	2010	L8
JQ663540	10-10JX	Jiangxi	2010	L8
JQ326271	WUH4	Hubei	2011	L8
JQ309823	GX10-48	Guangxi	2010	L8
JQ309822	GX10-42	Guangxi	2010	L8
JQ308798	QYYZ	Guangdong	2011	L3
JN864948	DY	Guangdong	2007	L5
JN836553	SCwhn09CD	Sichuan	2009	L8
JN662424	GM2	Guangdong	2011	L3
JN387271	GDQY1	Guangdong	2007	L8
JN256115	SD0901	Shandong	2009	L8
JF800911	ZCYZ	Shandong	2009	L8
JF796180	FS	Guangdong	2010	L8
JF748718	DC	na	2010	L8
JF748717	YD	na	2009	L8
JF268684	09HEN1	Henan	2009	L8
JF268683	09HUB2	Hubei	2009	L8
JF268682	09HUB1	Hubei	2009	L8
JF268681	09DB2	na	2009	L8
JF268680	09HEN2	Henan	2009	L8
JF268679	09HEB	Hebei	2009	L8
JF268678	09SD	Shandong	2009	L8
JF268677	09DB1	na	2009	L8
JF268676	09BJ	Beijing	2009	L8
JF268675	09JS	Jiangsu	2009	L8
JF268674	09HUN2	Hunan	2009	L8
JF268673	09HUN1	Hunan	2009	L8
JF268672	09SC	Sichuan	2009	L8
HQ843181	SX-09	Shanxi	2009	L8
HQ843180	SD-09	Shandong	2009	L8
HQ843179	HLM-09	Henan	2009	L8
HQ843178	HLJ-09	Henan	2009	L8
HQ416720	TA-12	Shandong	2008	L8
HQ401282	Shaanxi-2	Shanxi	2007	L8
HQ315837	SY0909	Jiangsu	2009	L8
HQ315836	NT0801	Jiangsu	2008	L8
HQ315835	BB0907	Guangxi	2009	L8
HM853673.2	WUH3	Hubei	2008	L8
HM214915	GX09-32	Guangxi	2009	L8
HM214914	GX09-29	Guangxi	2009	L8
HM214913	GX09-16	Guangxi	2009	L8
HM189676	HLJHL	Heilongjiang	2009	L8
HM016159	ZP-1	Shandong	2009	L8
HM016158	JN-HS	Shandong	2008	L8
HM011104	BJSY07	Beijing	2007	L8
GU461292	AH0701	Anhui	2007	L8
GU454850	GDQY2	Guangdong	2007	L8
GU269541	GD3	Guangdong	2005	L8
GU232738	YN9	Yunnan	2008	L8
GU232735	KP	na	2008	L8
GU169411	08HuN	Hunan	2008	L8
GU168569	08SDWF	Shandong	2008	L8
GU168568	09HUB5	Hubei	2009	L8
GU168567	09HUB7	Hubei	2009	L8
GU143913	GDp100	Guangdong	2009	L8

GQ914997	SD1-100	Shandong	2009	L1
GQ857656	SX-1	Shanxi	2009	L8
GQ374442	GDBY1	Guangdong	2008	L8
GQ374441	GDQJ	Guangdong	2007	L8
GQ359108	SD-CXA/2008	Shandong	2008	L8
GQ351601	BJ0706	Beijing	2007	L8
GQ330474	APRRS	Shanghai	2009	L7
FJ950747	BJSD	Beijing	2007	L8
FJ950746	BJPG	Beijing	2007	L8
FJ950745	BJBLZ	Beijing	2007	L8
FJ950744	BJSY-1	Beijing	2007	L8
FJ895329	SX2009	Shanxi	2009	L8
FJ889130	CWZ-1-F3	na	2008	L8
FJ889129	CBB-1-F3	na	2008	L8
FJ797690	HN-HW	Hunan	2006	L8
FJ536165	NB/04	Zhejiang	2004	L8
FJ393459	07BJ	Beijing	2007	L8
FJ393458	07HEBTJ	Hebei	2007	L8
FJ393457	07HEN	Henan	2007	L8
FJ393456	07NM	Inner Mongolia	2007	L8
FJ175689	PRRSV03	Gansu	2008	L5
FJ175688	PRRSV02	Gansu	2008	L5
FJ175687	PRRSV01	Gansu	2008	L5
EU939312	JSyx	Jiangsu	2006	L8
EU880443.3	GS2004	Gansu	2004	L5
EU880442.2	GS2003	Gansu	2003	L5
EU880441.2	GS2002	Gansu	2002	L5
EU880440	CH2003	na	2003	L8
EU880439	CH2004	na	2004	L8
EU880438.1	CH2002	na	2002	L8
EU880437.2	HN2007	Hunan	2007	L8
EU880436	XL2008	na	2008	L8
EU880435.2	YN2008	Yunnan	2008	L8
EU880434.2	SX2007	Shanxi	2007	L8
EU880433.2	GD2007	Guangdong	2007	L8
EU880432.2	JX2006	Jiangxi	2006	L8
EU880431.2	GS2008	Gansu	2008	L8
EU864233	TP	na	2006	L8
EU864232	SHB	na	2005	L8
EU864231	CG	na	2007	L8
EU860249	NM1	Inner Mongolia	2007	L8
EU860248	TJ	Tianjin	2006	L8
EU825724	GD	Guangdong	2007	L8
EU825723	BJ	Beijing	2007	L8
EU708726	JX143	Jiangxi	2006	L8
EU678352	WUH2	Hubei	2008	L8
EU624117	XH-GD	Guangdong	2007	L8
EU262603	Em2007	Hubei	2007	L8
EU236259	HPBEDV	na	2007	L8
EU200962	Henan-1	Henan	2007	L8
EU200961	Jiangxi-3	Jiangxi	2007	L8
EU187484	WUH1	Hubei	2007	L8
EU144079	SY0608	Jiangsu	2006	L8
EU109503	GD	Guangdong	2006	L8
EU109502	LN	Liaoning	2006	L8
EU106888	SHH	Shanghai	2006	L8
EU097707	BJsy06	Beijing	2006	L8
EU097706	NX06	Ningxia	2006	L8
EF641008	JXwn06	Jiangxi	2006	L8
EF635006	HUN4	Hunan	2006	L8
EF517962	HuN	Hunan	2007	L8
EF153486	CC-1	Jilin	2005	L5
EF112447	HEB1	Hebei	2006	L8
EF112446	HUB2	Hubei	2006	L8
EF112445	JXA1	Jiangxi	2006	L8
EF075945	HUB1	Hubei	2006	L8
DQ459471	S1	na	2006	L5
AY457635	HN1	Hunan	2003	L5
AY262352	HB-2(sh)/2002	Hebei	2002	L8
AY150312	HB-1(sh)/2002	Hebei	2002	L8
AY032626	CH-1a	Beijing	1996	L8
AF331831	BJ-4	Beijing	1997	L5

Table S2 Recombination information of PRRSV-2 in China from 2019 to 2023.

Accession number	Strain	Lineage	Year	Major parent	Minor parent	Region 1	Region 2	Region 3	Region 4
OQ986590.1	SCABTC-202301	L8	2023	L8	L1	11002–12100			
OQ986591.1	SCABTC-202302	L1	2023	L1	L8	12190–15106			
OR753369.1	CHNMGKL1-2304	L1	2023	L1	L5	13074-13815			
OR766560.1	SCABTC-202309	L1	2023	L1	L8	1-616	1464-2124	7636-8708	9237-9314
OR711915.1	GD-7	L1	2023	L1	L8	671-2154	5726-6970	7006-8132	
OR662185.1	BDSP-1	L1	2023	L1	L8	1-2212	5955-9660	14638-14852	
OR575928.1	HN-NY/2023	L1	2023	L1	L8	62-1495	5834-8689		
OR582383.1	GX-3	L1	2023	L1	L8	7775-9335			
OR826316.1	TZJ3116	L1	2023	L1	L3	7236-8638			
OR826315.1	TZJ3115	L1	2023	L1	L3	7236-8166			
OR826314.1	WK730	L1	2023	L1	L8	7328-8571			
OR826313.1	TZJ3005	L1	2023	L1	L8	7260-8582			
OR269980.1	2023GD-4	L1	2023	L1	L5	14716-15029			
OR365672.1	SCABTC-202305	L1	2023	L1	L8	8198-9308	12286-12770		
OR365675.1	SCABTC-202308	L1	2023	L1	L5	13266-13796			
OR670493.1	SCCD22	L1	2022	L1	L8	1146-3577	11457-12986		
OR800933.1	hy_2203	L1	2022	L1	L8	5738-8605			
OQ538074.1	SDYT91	L8	2022	L8	L1	14604-14626	15828-16010		
OR369723.1	GZ2022	L1	2022	L1	L8	1-1950	6184-7294	8209-9400	11456-13426
OR146747.1	SF5	L1	2022	L1	L8	658-2142	6636-7249	11485-13270	
OR146748.1	SF7	L1	2022	L1	L8	658-2140			
OR146749.1	ZH12	L8	2022	L8	L3	1-1050	1704-4135	16022-16136	
OP716076.1	CH-HNPY-01	L1	2022	L1	L8	5823-6886	7408-8657		
ON142049.1	PRRSV-HQ-2020	L8	2022	L8	L1	9430-11472	15314-16129		
OR250810.1	HuN-ZZ	L8	2022	L8	L5	653-1350	4227-4804		
OP734317.1	CHbj2102	L1	2022	L1	L3	11985–13226			
OP734318.1	CHbj2103	L1	2022	L1	L3	11984–13531			
OP734316.1	CHbj2101	L1	2022	L1	L8	1313–1813	5688–6587	6848–8882	
OP131596.1	HBag-4	L1	2022	L1	L8	126–538	1858–2002		
OP764591.1	GSWW	L8	2022	L1	L8	5634–11983			
OQ748875.1	TZJ2756	L1	2022	L1	L5	14072-14596			
OQ459662.1	GXBY20220301	L1	2022	L1	L8	5390–6958	7380–8890		
OQ459664.1	GXGL20220301	L1	2022	L1	L8	5422–6958	7380–8890		
OQ459666.1	GXYL20220501	L1	2022	L1	L8	1–486	5688–6712	15528–15566	
OQ790147.1	WK621	L1	2022	L1	L8	5416–6259	6976–8172	14328–14532	
OQ790146.1	TZJ2451	L1	2022	L1	L8	274–1998	5406–7754	14349–14483	
OQ459667.1	GXYN20220502	L8	2022	L8	L3	5806–6206	11202–12930	12937–13101	
OQ459668.1	GD20220303	L8	2022	L8	L1	2002–4236	12254–13940	15100–15566	
OQ506516.1	SDWH86	L8	2022	L8	L1	1–45	13785–15566		
OP805381.1	SDLY27-2022	L8	2022	L8	L1	12267–12794	13346–13876		
OR800932.1	sg_2107	L1	2021	L1	L8	1-2138	5917-9476	10516-10923	13276-13582
OR800931.1	zq_2109	L8	2021	L8	L1	6509-8743	11458-12490		
OR800930.1	qy_2104	L1	2021	L1	L3	12682-13357	13401-13777		
OR800927.1	sg_2108	L8	2021	L8	L3	1-1038	15926-16085		
OR800925.1	qy_2105	L1	2021	L1	L3	12640-13315	13359-13726		
OR800926.1	sg_2104	L8	2021	L8	L3	1-1038	15939-16085		
OR468246.1	GXNN20210906	L3	2021	L3	L8	304-1637	1714-2636		
OQ817851.1	PRRSV/TZJ1712	L8	2021	L8	L1	2298-4414	14424-16074		
OQ871558.1	HN-1	L1	2021	L1	L8	11477-13018			
OM202897.1	GX4852	L1	2021	L1	L8	1–2002	5832–8142	8300–8948	
OM293962.1	HN0713	L1	2021	L1	L8	1–1950	5934–8912	11345–11601	
OP784963.1	HuN-XT-B	L1	2021	L1	L8	1–1039	7694–8720		
OM202899.1	GX5416	L8	2021	L8	L1	2014–3602			
OM293959.1	GDGZ0408	L8	2021	L8	L3	8803–9622	12831–13440	13955–14957	
OM293960.1	GDHY0425	L8	2021	L8	L5	5021–5344	9811–12003	15200–15411	
ON691480	GD-F1	L1	2021	L1	L1	6124–6535	11688–12778	13305–14614	
ON691479	GD-H1	L1	2021	L1	L1	11722–12778	13471–14033		
OK486522	GXFCG20210401	L1	2021	L1	L8	693–2034			
OK486523	GXQZ20210403	L1	2021	L1	L8	672–1332	5394–7933		
OK486524	GXNN20210506	L1	2021	L1	L8	1–504	695–2018	7934–8890	
OL310959	CN/FJGD01/2021	L1	2021	L1	L8	5489–6190	6905–8179		
OL422841	CN/N2	L1	2021	L1	L8	1156–1495	7684–10183	12752–13038	
OL422842	CN/N0	L1	2021	L1	L8	7210–7908	7914–8861		
OL422844	CN/Z0	L1	2021	L1	L8	7345–7493	8004–8266		
OL516348	HLJPY32-2109	L1	2021	L1	L8	5604–6432			
OL516353	HLJTZJ1988-2106	L1	2021	L1	L8	5831–6432	6853–8150	8322–8944	
OL516354	HLJTZJ2007-2106	L1	2021	L1	L8	6857–9072			
OL516355	HLJTZJ2090-2107	L1	2021	L1	L8	1499–1813			
OL516356	HLJTZJ2165-2108	L1	2021	L1	L8	6857–8170			
OL516359	JLTZJ2050-2107	L1	2021	L1	L8	7786–8930			
ON180781	YC-2020	L1	2021	L1	L8	5372–8180	14326–14530		

ON462045	GXTZJ2325-2112	L3	2021	L3	L1	6826–7469	11138–11570		
MZ712110.1	HB2104	L1	2021	L8	L1	1765–5953			
OL416130	CN/L3/2021	L8	2021	L8	L1	479–701	1848–5504		
ON093974.1	CN/SS1/2021	L8	2021	L8	L1	479–701	1840–2214	3902–5340	
OL422843	CN/Q9	L8	2021	L8	L5	12064–15528			
OR518274.1	SXht2012	L1	2020	L1	L8	1-1666	5828-6888		
OR102498.1	SH	L1	2020	L1	L8	13275-13458	14492-14720		
OR066233.1	HuBXW	L1	2020	L1	L8	360-1652	5844-8692		
OP168793.1	SDHY_DZ037	L1	2020	L1	L1	6515–10323	10916–11773	12543–12665	
OM202894.1	GX505	L1	2020	L1	L8	1–2002	5832–8142	8300–8948	
OM202895.1	GX1858	L1	2020	L1	L8	1–2002	5834–8142	8302–8910	
OM202902.1	GX11045	L8	2020	L3	L8	1–11336			
OM202903.1	GX11373	L8	2020	L8	L3	2180–4070	13778–14414		
OM293961.1	GDYJ1224	L1	2020	L1	L8	422–1121	5921–8097	12212–13165	
OL771206	CH/SCCD-4/2020	L1	2020	L1	L3	1605–4050			
MW079495	2020-Acheng-1	L1	2020	L1	L5	12494–13356			
MW531679	GXNN202004a	L1	2020	L1	L8	1–446	5721–6902		
MW561594	GXNN202004	L1	2020	L1	L8	1–2024	5812–8964	11356–11561	8803–8950
MW803134	SCcd2020	L1	2020	L1	L8	4084–4586	5394–6431	6815–8158	
MZ047780.1	BJ20-06	L1	2020	L1	L8	5422–8179			
MZ047781.1	SDlz20-04	L1	2020	L1	L8	5422–8179	14354–14554		
MZ342900	JS2020	L1	2020	L1	L8	5445–8239	12728–13000	14323–14525	
MZ747442.1	CH/SCMY-3	L1	2020	L1	L8	1–1547	11035–11570		
MZ747443.1	CH/SCNC-2	L1	2020	L1	L8	544–1740			
MZ747444.1	CH/SCPZ	L1	2020	L1	L8	5403–6217	6851–8094		
OL516347	HLJPY18-2009	L1	2020	L1	L8	693–1234			
MZ160905.1	PRRSV NA	L1	2020	L1	L8	5380–6422	6835–8103		
OL771209	CH/SCYB-2/2020	L1	2020	L1	L8	14191–14346			
ON462044	HNTZJ1714-2011	L1	2020	L5	L1	491–4000			
MW408254	SD 2020	L5	2020	L5	L8	9858–11104	11125–11408		
OL422822	CN/L4/2020	L8	2020	L8	L1	1840–5504			
OL422837	CN/F3	L8	2020	L8	L1	8149–8300			
OL422838	CN/H2	L8	2020	L8	L1	12035–14051			
OL422839	CN/H5	L8	2020	L8	L1	13740–15522			
ON365556.1	CN/SS0/2020	L8	2020	L8	L1	529–701	2154–2206	4094–5506	
OL416129	CN/I9/2018	L1	2019	L1	L3	12131–12767			
OL771205	CH/SCMY-2/2019	L1	2019	L1	L5	12475–12885			
OM201178.1	S130	L1	2019	L1	L5	7874–8104			
OM201180	S145	L1	2019	L1	L5	7971–8104			
MT165636	GD1909	L1	2019	L1	L8	1–1303	4395–5984	7821–8134	
MZ399800	NL1207	L1	2019	L1	L8	5403–6210	6852–8128		
OL416127	CN/G9/2018	L1	2019	L1	L8	695–2002			
OL422823	CN/N4/2019	L1	2019	L1	L8	7342–7767	7910–8278	8370–9031	
OL422836	CN/F2	L1	2019	L1	L8	5780–6217	6851–8890		
OL422840	CN/J2	L1	2019	L1	L8	342–811	6262–7493	8204–8456	
OM201179.1	S136	L1	2019	L1	L8	5940–6317	6761–8118		
MT075480	SC/DJY	L1	2019	L1	L8	7868–8944	11095–11561	11864–12343	
OM201192.1	H64	L1	2019	L1	L8	8798–8882			
OM201193.1	HB94	L1	2019	L1	L8	5496–8132			
ON462043	HNLCL15-1903	L1	2019	L1	L8	1–1662	11035–11570	13067–13294	
MN547966	JSTZ1904-664	L8	2019	L8	L1	11040–12067			
MN547967	JSTZ1907-714	L8	2019	L8	L1	11047–12084			
MT316312	HB18-4	L8	2019	L8	L1	11570–12034			
MT780871	JSYZ1909-16	L8	2019	L8	L1	11098–12034			
MZ219271.1	CH/GX/2475	L8	2019	L8	L1	13846–14598	14834–15391		
MZ219272	CH/GX	L8	2019	L8	L1	13863–14598	15100–15566		
OL422833	CN/C2	L8	2019	L8	L1	10987–12084			
OL422834	CN/L1	L8	2019	L8	L1	11018–12067			
OL422835	CN/L2	L8	2019	L8	L1	1712–3566	8159–8300	10987–12084	
OM949993.1	GDqy-1909	L8	2019	L8	L1	11138–12084			
MN119309	XJ1904-39	L1	2019	L8	L1	2122–5756	12281–15012		
MZ322956	SDRZ01	L8	2019	L8	L3	586–952			
OM201185.1	G101	L8	2019	L8	L5	12400–12596	12803–15542		

Table S3 Primers used for amplification of the genome of PRRSV.

Primers	Sequence (5′ − 3′)	Use
A-F	GGTACCGGGCCCCCCctcgagTTAATTAAATTTAGGTGA	Amplified A fragment
A-R	AGCTCCACCGCGGTGgcggccgccaattgGACAGTGAG	Amplified A fragment
B-F	CTGACCGCCTTCTCACTGTCcaattgCTATTACCCT	Amplified B fragment
B-R	AGCTCCACCGCGGTGgcggccgcggatccAGAATCGCCACACGCGGTGAAGCAGAA	Amplified B fragment
C-F	GCGTGTGGCGATTCTggatccCCAGTGATTACCGAA	Amplified C fragment
C-R	AGCTCCACCGCGGTGgcggccgcatcgatCGCAGGACGCAAGATCAGCTTCAA	Amplified C fragment
D-F	ATCTTGCGTCCTGCGATCGATCCACACCTGCAATTGT	Amplified D fragment
D-R	AGCTCCACCGCGGTGGCGGCCGCGGCGCGCCCGAAACGCATCATTGTAATCCTCCCAGT	Amplified D fragment
E-F	GATTACAATGATGCGTTTCGggcgcgccAGAAAGGGAAAATTT	Amplified E fragment
E-R	AGCTCCACCGCGGTGgcggccgcacgcgtGGTTATCATTTGCCGCAATCG	Amplified E fragment
F-F	GATTGCGGCAAATGATAAggacgcgtTTGTCGTCCGGCGTCCCGGCT	Amplified F fragment
F-R	AGCTCCACCGCGGTGgcggccgcatttaaatTTTTTTTTTTTTTTTTTTTTTTTTTTTTTTTTAATTACGGCC	Amplified F fragment

The lowercase letters are the sequences of the restriction sites in the PRRSV genome, The capital letters are the sequence of PRRSV genome.
